# TNF hampers intestinal tissue repair in colitis by restricting IL-22 bioavailability

**DOI:** 10.1038/s41385-022-00506-x

**Published:** 2022-04-05

**Authors:** Justus Ninnemann, Caroline Winsauer, Marina Bondareva, Anja A. Kühl, Laura Lozza, Pawel Durek, Donata Lissner, Britta Siegmund, Stefan H. E. Kaufmann, Mir-Farzin Mashreghi, Sergei A. Nedospasov, Andrey A. Kruglov

**Affiliations:** 1 German Rheumatism Research Center (DRFZ), a Leibniz Institute, Berlin, Germany; 2 Belozersky Institute of Physico-Chemical Biology and Faculty of Bioengineering and Bioinformatics, M.V. Lomonosov Moscow State University, Moscow, Russia; 3 iPATH.Berlin, Core Unit of Charité-Universitätsmedizin Berlin, Charité – Universitätsmedizin Berlin, corporate member of Freie Universität Berlin, Humboldt-Universität zu Berlin, and Berlin Institute of Health, Berlin, Germany; 4 Department of Immunology, Max Planck Institute for Infection Biology, Berlin, Germany; 5 Department of Gastroenterology, Infectious Diseases and Rheumatology, Campus Benjamin Franklin, Charité – Universitätsmedizin Berlin corporate member of Freie Universität Berlin and Humboldt-Universität zu Berlin, Berlin, Germany; 6 Center for Precision Genome Editing and Genetic Technologies for Biomedicine, Engelhardt Institute of Molecular Biology, Russian Academy of Sciences, Moscow, Russia; 7 Institute of Cell Biology and Neurobiology, Charité – Universitätsmedizin Berlin, Berlin, Germany; 8 Belozersky Institute of Physico-Chemical Biology and Biological Faculty, M.V. Lomonosov Moscow State University, Moscow, Russia

## Abstract

Successful treatment of chronic inflammatory diseases integrates both the cessation of inflammation and the induction of adequate tissue repair processes. Strikingly, targeting a single proinflammatory cytokine, tumor necrosis factor (TNF), induces both processes in a relevant cohort of inflammatory bowel disease (IBD) patients. However, the molecular mechanisms underlying intestinal repair following TNF blockade during IBD remain elusive. Using a novel humanized model of experimental colitis, we demonstrate that TNF interfered with the tissue repair program via induction of a soluble natural antagonist of IL-22 (IL-22Ra2; IL-22BP) in the colon and abrogated IL-22/STAT3-mediated mucosal repair during colitis. Furthermore, membrane-bound TNF expressed by T cells perpetuated colonic inflammation, while soluble TNF produced by epithelial cells (IECs) induced IL-22BP expression in colonic dendritic cells (DCs) and dampened IL-22-driven restitution of colonic epithelial functions. Finally, TNF induced IL-22BP expression in human monocyte-derived DCs and levels of IL22-BP correlated with TNF in sera of IBD patients. Thus, our data can explain how anti-TNF therapy induces mucosal healing by increasing IL-22 availability and implicates new therapeutic opportunities for IBD.

## Introduction

Inflammatory bowel disease (IBD) is a chronic autoimmune disease of the gastrointestinal tract driven by an aberrant immune response towards microbial constituents in genetically susceptible hosts^1,2^. IBD incidence has increased worldwide profoundly over the past decades with no curative treatment currently being available. Notably, even in clinical remission stage, mucosal healing is achieved in less than half of the IBD patients, leaving the possibility of subsequent bacterial translocation, further colonic epithelial damage, and clinical relapse. Various therapies are being applied in clinical practice, but in most of the cases, they are directed towards cessation of inflammation, without actively influencing tissue repair processes.

Interestingly, TNF blockade, one of the biologic therapies approved for IBD treatment, may also result in mucosal tissue repair in a significant fraction of patients^3–6^. TNF is a pleiotropic cytokine exhibiting both protective and pathogenic functions in vivo^7^. Its chronic overproduction is observed in many autoimmune diseases, such as rheumatoid arthritis, ankylosing spondylitis and IBD^8,9^. Therapeutic strategies based on TNF neutralization are highly efficient against these autoimmune diseases and can significantly decrease inflammation^8^.

TNF exhibits pleiotropic functions during intestinal inflammation. For instance, it induces production of chemokines that recruit proinflammatory myeloid cells to the colon^10^. Also, TNF controls tissue barrier functions by regulating apoptosis of intestinal epithelial cells (IECs), expression of tight junction proteins and mucus secretion^11–13^. Thus, anti-TNF therapy based on various antibodies neutralizing TNF was successfully implemented as treatment option for IBD. Apart from direct TNF neutralization, such antibodies may bind to the transmembrane form of TNF (tmTNF) expressed by monocytes and T cells and promote their depletion via antibody-induced cytotoxicity^14^. Consistent with this, a positive therapeutic response to anti-TNF therapy correlates with increased frequency of tmTNF-producing cells at sites of inflammation in the colon^15^. Despite the plethora of data on the action of TNF on various target cells, the precise in vivo action of TNF during active colitis remains scarce.

IL-22 is a cytokine produced by various immunocytes, such as type III innate lymphoid cells (ILC3), T cells and neutrophils^16^. The IL-22 receptor is exclusively expressed on cells of non-hematopoietic origin, such as IECs, and this receptor engagement results in cell proliferation and tissue repair upon insult. If not properly controlled, IL-22 signaling may also induce malignancy^17^. A soluble antagonist of IL-22 (IL-22BP) was described that may neutralize biological effects of IL-22 in vivo and, thereby, counteract the tissue repair processes^18^. Interestingly, IL-22BP expression is increased during IBD^19,20^, and correlation between mRNA levels of TNF and IL-22BP in colonic tissues of IBD patients has been observed^20^. However, the relationship between IL-22 and TNF in tissue repair remains elusive.

Here, we show that enhanced levels of TNF during intestinal inflammation induce the expression of soluble IL-22BP in the colon and, thereby, antagonize IL-22/STAT3-mediated mucosal repair during colitis Table 1. Pharmacological blockade of TNF produced by T cells only (T-TNF) resulted in reduced inflammation and dampened colonic TNF production. This led to diminished colonic IL-22BP expression, increased bioactive IL-22 abundance, followed by enhanced intestinal IECs proliferation, and restoration of colonic epithelial functions. Finally, we found that TNF produced by IECs controls IL-22BP production during colitis. Thus, our data demonstrate that anti-TNF therapy induces mucosal healing by increasing IL-22 bioavailability and implicate new therapeutic strategies for IBD treatment in humans.

**Table 1:** Monoclonal antibodies used for immunofluorescence staining (cell surface).

Antibody	Conjugate	Clone	Company	Identifier
B220	Biotin	RA3.6B2	DRFZ facility	N/A
CD103	PE	2E7	eBioscience	Cat # 12-1031-82
CD11c	Biotin	N418	DRFZ facility	N/A
CD25	APC	pC61.5	eBioscience	Cat # 17-0251-82
CD25	Biotin	pC61.5	DRFZ facility	N/A
CD4	PE-Cy7	RM4-5	eBioscience	Cat # 25-0042-82
CD45	FITC	30-F11	eBioscience	Cat # 11-0451-82
CD45RB	PE	16 A	eBioscience	Cat # 12-0455-82
CD8α	Biotin	53-6.72	DRFZ facility	N/A
Gr1	Cy5	RB6-8C5	DRFZ facility	N/A
Ly6C	PE-Cy7	HK1.4	eBioscience	Cat # 25-5932-82
CD11b	eFluor 450	M1/70	eBioscience	Cat # 48-0242-82
TCRβ	PE	H57-597	eBioscience	Cat # 12-5961-82
MHCII	PE	M5/114	DRFZ facility	N/A
Ly6G	PE	1A8-Ly6G	Thermofisher Scientific	Cat # 12-9668-82
EpCAM1	APC	G8.8	Thermofisher Scientific	Cat # 17-5791-82

## Results

### T-cell derived TNF perpetuates disease progression in a humanized mouse, TNF-driven model of colitis

**Fig. 1:**
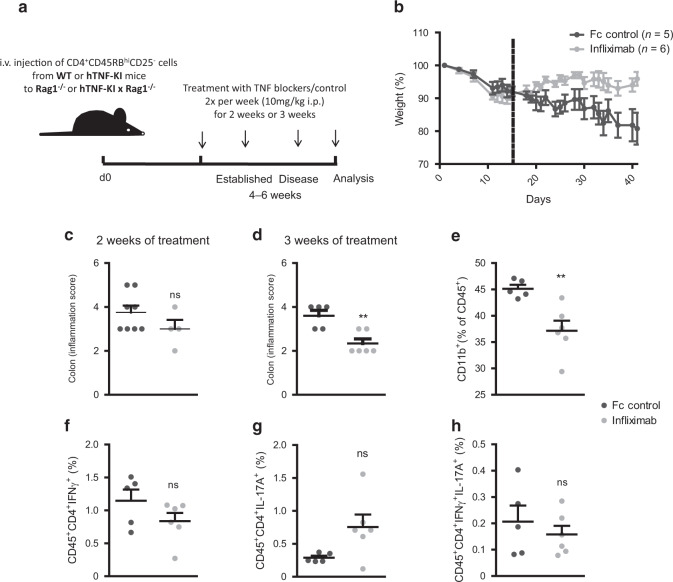
Inhibition of colitis by human TNF blockade in humanized TNF model of colitis. **A** Humanized TNF colitis model. Rag1^−/−^ or hTNF‐KI x Rag1^−/−^ mice were reconstituted with naive T cells from either WT or hTNF‐KI mice. Mice were treated once they had lost > 5 % of their initial weight twice per week for two or three weeks with various anti-TNF agents or respective controls (10 mg/kg; i.p.). **B** Weight of hTNF‐KIxRag1^−/−^ mice reconstituted with naive T cells from hTNF‐KI mice and treated with infliximab (*n* = 6) or Fc control (*n* = 5) (10 mg/kg) twice per week. Data are representative of two independent experiments. **C** Colitis inflammation scores of hTNF‐KIxRag1^−/−^ mice reconstituted with naive T cells from hTNF‐KI mice upon 2 weeks treatment with infliximab (*n* = 4) or isotype control (*n* = 8). Data are representative of two independent experiments. **D** Colitis inflammation scores of hTNF‐KIxRag1^−/−^ mice reconstituted with naive T cells from hTNF‐KI mice upon 3 weeks treatment with infliximab (*n* = 6) or isotype control (*n* = 5). Frequencies of CD11b^+^ (CD45^+^CD11b^+^) **E**, Th1 (CD45^+^CD4^+^TCRβ^+^IFNγ^+^) **F**, Th17 (CD45^+^CD4^+^TCRβ^+^IL-17A^+^) **G** and Th1/17 (CD45^+^CD4^+^TCRβ^+^IL-17A^+^IFNγ^+^) **H** cells after 3 weeks of infliximab (*n* = 6) or Fc control treatment (*n* = 5). All data in **D**–**H** are representative of two independent experiments. Data represent mean values ± SEM. *p < 0.05, **p < 0.01, ***p < 0.001, as calculated by Student’s t‐test, ns not significant.

Mouse studies aimed to address the role of TNF in autoimmune colitis mostly rely on reverse genetic approaches. However, in conventional knockout mice gene function is ablated both at disease onset and during disease progression. In contrast, IBD patients typically present a full-blown disease at the initiation of treatment and thus anti-TNF therapy is generally applied after this stage. Furthermore, clinical treatment involves a range of specific anti-human TNF (anti-hTNF) agents with distinct pharmacological and physico-chemical characteristics that cannot be evaluated in available mouse models. Therefore, we developed a novel humanized mouse, TNF-driven model of autoimmune colitis that allows studying TNF inhibition by all clinically used anti-hTNF drugs at the stage of fully developed colitis (Fig. 1). To this end, we utilized a human TNF knock-in (hTNF-KI) mouse, in which the human TNF gene with its promoter has been inserted instead of the murine counterpart (Fig. S1A)^21^. To generate a mouse colitis model dependent solely on human TNF expression, we transferred naive T cells isolated from hTNF-KI donor animals into offspring of hTNF-KI mice crossed with Rag1^−/−^ mice (Fig. 1A)^21,22^. To dissect the contribution of T cell-derived TNF versus non-T cell-derived TNF, we performed transfers of T cells from hTNF-KI mice to Rag1^−/−^ recipients or WT T cells to hTNF-KI×Rag1^−/−^ mice (Figs. 1A and S1B, C). The former setting allows specific blockade of T cell-derived TNF by anti-hTNF agents, whereas in the latter case the application of hTNF blockers depletes TNF from the non-T cell compartments.

In the fully “humanized TNF” colitis model, when naive T cells from hTNF-KI mice were transferred into hTNF-KI×Rag1^−/−^ recipients, chronic autoimmune colitis developed (Fig. 1B). Once mice lost 5% of their initial weight, they were blindly divided into two groups and treated either with infliximab, a chimeric antibody that binds only human, but not mouse TNF^23^, or with irrelevant human Fc control antibody (Fig.1B–H). Interestingly, mice started to gain weight several days after initiation of treatment with infliximab (Fig. 1B). Further histological analysis revealed that colonic inflammation was significantly reduced after 3 weeks of anti-TNF therapy, whereas 2 weeks of treatment failed to ameliorate inflammation despite weight gain (Fig. 1C, D). Reduction of colonic inflammation was accompanied with diminished CD11b^+^ cells in the colon upon anti-TNF therapy (Fig. 1E), whereas the T cell compartment was unaffected (Fig. 1F–H).

**Fig. 2:**
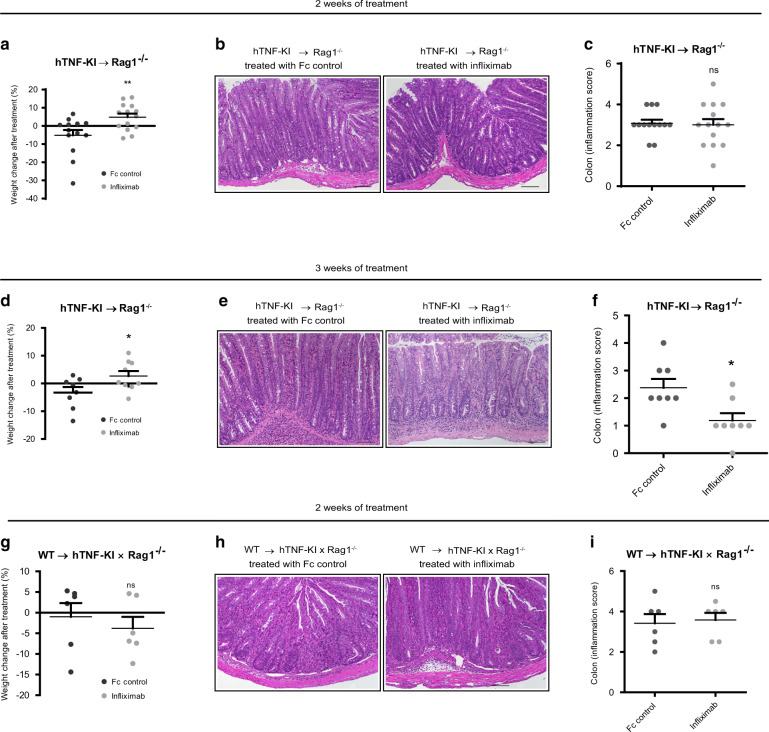
T cell-derived TNF is pathogenic during established colitis. Naive hTNF-KI T cells were transferred to Rag1^−/−^ recipients, anti-TNF (infliximab; 10 mg/kg; i.p. twice per week) was administered once mice had lost 5% of their initial weight. **A** Weight changes 2 weeks after treatment of colitic mice with either Fc control or infliximab (both 10 mg/kg; i.p, twice per week). Representative images of Hematoxylin/Eosin stained tissue sections **B** and inflammation score **C** of the colon in mice treated with either Fc control of infliximab for 2 weeks. Scale bar is equal to 100 µm. **A**–**C** Data from three independent experiments are shown. **D** Weight changes 3 weeks after treatment of colitic mice with either Fc control or infliximab (both 10 mg/kg; i.p, twice per week). Representative images of Hematoxylin/Eosin stained tissue sections **E** and inflammation score **F** of the colon in mice treated with either Fc control of infliximab for 3 weeks. Scale bar is equal to 100 µm. **D**–**F** Data from two independent are shown. Naive WT T cells were transferred to hTNFK-KI×Rag1^−/−^ recipients, anti-TNF (infliximab; 10 mg/kg; i.p. twice per week) was administered once mice had lost 5% of their initial weight. **G** Weight changes 2 weeks after treatment of colitic mice with either Fc control or infliximab (both 10 mg/kg; i.p, twice per week). Representative images of Hematoxylin/Eosin stained tissue sections **H** and inflammation score (**I**) of the colon in mice treated with either Fc control or infliximab for 2 weeks. Scale bar is equal to 100 µm. Data are representative of two independent experiments. Data represent mean values ± SEM. *p < 0.05, **p < 0.01, ***p < 0.001, as calculated by Student’s t-test; ns not significant.

Both the transfer of hTNF-KI naive T cells into Rag1^−/−^ recipients and of WT naive T cells into Rag1^−/−^ and hTNF-KI×Rag1^−/−^ mice induced chronic colitis (Figs. S2, S3). Comparative analysis revealed that kinetics of weight loss, histopathological score, inflammatory cell composition, EC proliferation and apoptosis of epithelial cells in the colon were similar to disease induced by transfer of naive WT T cells in Rag1^−/−^ mice (Figs. S2, S3). Blockade of T cell-derived TNF (T-TNF) significantly increased the weight of mice after two weeks of treatment (Fig. 2A). However, this was not reflected by colon histopathology when compared with Fc control treated animals (Fig. 2B, C), indicating that two weeks or less of treatment were insufficient for amelioration of clinical signs of inflammation quantified by histopathology. When T-TNF was blocked for 3 or more weeks, both weight loss and inflammation were reversed (Fig. 2D, E, F). In contrast, blockade of non-T cell-derived TNF using anti-hTNF therapy in either short-term or long-term modality did not affect the course of disease (Figs. 2G, H, I and S4). Interestingly, T-TNF was reported to have slight effects on colitis severity^24^. Given the complex interaction of microbiota and immune system in the induction of chronic colitis, we hypothesized that microbiota composition may influence TNF expression by colonic cells and subsequent involvement of T-TNF in colitis development. To address this, we induced colitis by transferring of naive hTNF-KI T cells into Rag1^−/−^ recipients and modified the microbiota in these mice by vancomycin treatment concomitantly with anti-TNF treatment (Fig. S5). Interestingly, vancomycin treatment alone improved the weight of colitic mice and reduced human TNF production by T cells, but not by other cells (Fig. S5 and data not shown). Subsequently, myeloid cell numbers migrating to the colon diminished upon Vancomycin application (Fig. S5). Importantly, infliximab treatment, once applied together with vancomycin, did not further improve disease course. Thus, microbiota composition during colitis may serve as a definitive factor in T-TNF dependency of colitis.

Taking into account the previous reports on possible TNF-independent effects of infliximab in vivo^25^, we also analyzed the effect of infliximab on WT colitis: infliximab administration did not show any impact on disease progression and on colonic immune cells when colonic inflammation was induced in Rag1^−/−^ mice upon transfer of naive WT T cells (Fig. S6A–E). Finally, infliximab did not induce apoptosis of LPS-stimulated splenocytes in vitro (Fig. S6F), in contrast to published data^26^.

Thus, such experimental setup represents a clinically relevant model for studies of anti-hTNF drugs in vivo that allows dissecting the contribution of T versus non-T cell-derived TNF in fully developed “human-like” colitis and implies an important role of TNF produced by T cells in driving disease progression during colitis.

### T cell-derived TNF promotes inflammation via chemokine-mediated recruitment of myeloid cells

**Fig. 3:**
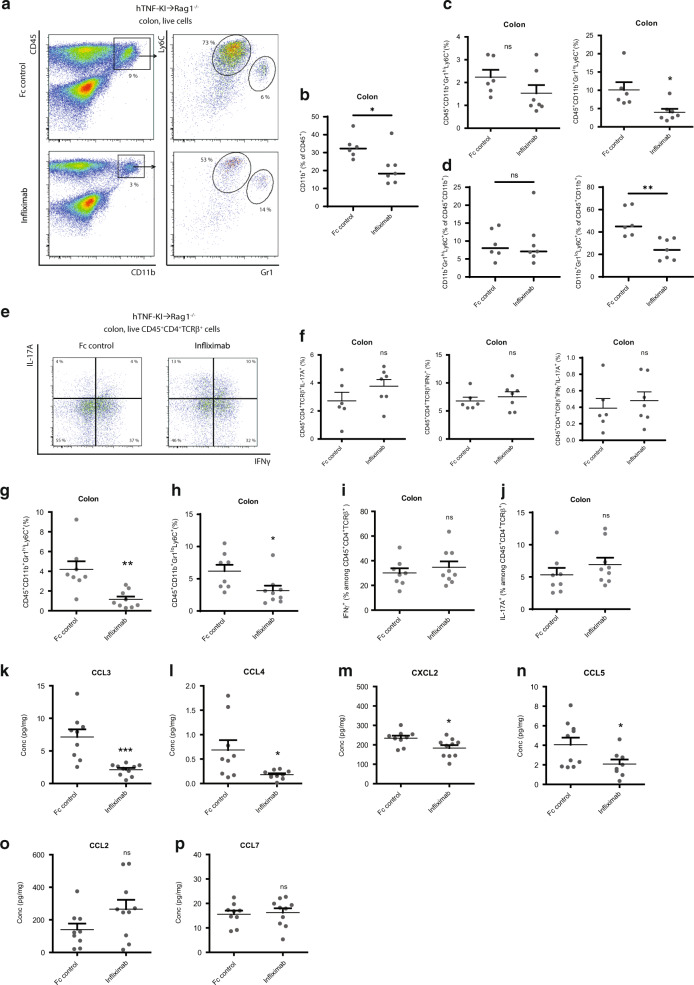
T-TNF drives disease via chemokine-mediated recruitment of myeloid cells. **A**–**D** Frequencies of granulocytes (CD45^+^CD11b^+^Gr1^high^Ly6C^+^) and inflammatory monocytes (CD45^+^CD11b^+^Gr1^low^Ly6C^+^) in the colon after 2 weeks of anti-TNF therapy. **E**, **F** Frequencies of Th1 (CD45^+^CD4^+^TCRβ^+^IFNγ^+^), Th17 (CD45^+^CD4^+^TCRβ^+^IL-17A^+^) and Th1/17 (CD45^+^CD4^+^TCRβ^+^IFNγ^+^IL-17A^+^) cells after 2 weeks of anti-TNF therapy. **A**–**F** Data are representative of three experiments are shown. **G**, **H** Frequencies of granulocytes (CD45^+^CD11b^+^Gr1^high^Ly6C^+^) and inflammatory monocytes (CD45^+^CD11b^+^Gr1^low^Ly6C^+^) in the colon after 3 weeks of anti-TNF therapy. **I**, **J** Frequencies of Th1 (CD45^+^CD4^+^TCRβ^+^IFNγ^+^) and Th17 (CD45^+^CD4^+^TCRβ^+^IL-17A^+^) cells after 3 weeks of anti-TNF therapy. Concentrations of CCL3 **K**, CCL4 **L**, CXCL2 **M**, CCL5 **N**, CCL2 **O**, CCL7 **P** in colonic explants after 2 week anti-TNF therapy as in **A**. **G**–**P** Data of two independent experiments are depicted. Data represent mean values ± SEM. *p < 0.05, **p < 0.01, ***p < 0.001, as calculated by Student’s t-test; ns not significant.

Since we observed disease amelioration upon T-TNF blockade, we next quantified colonic inflammatory infiltrates during the course of anti-TNF therapy. After two weeks of such therapy, we observed reduced numbers of inflammatory monocytes, but not of granulocytes, both in colon and MLN (Figs. 3A–D and S7A, B). Additionally, blockade of T-TNF did not affect overall T cell numbers, IL-17A-producing or IFNγ-producing T cell subsets and did not result in a significant increase of apoptosis of T cells (Figs. 3E, F and S7D). Inflammatory monocytes are recruited as Ly6C^+^MHCII^+^CD64^low^ cells to the colon during colitis, where they rapidly differentiate to inflammatory macrophages by upregulating MHCII and CD64^27^. MHCII expression on colonic monocytes was not affected by T-TNF blockade (Fig. S7E), further suggesting that the decrease in monocyte abundance was due to the inhibition of their recruitment and not differentiation.

Consistent with the inflammatory score, a prolonged infliximab administration in this setting resulted in significantly reduced numbers of both granulocytes and inflammatory monocytes in the colon as compared to Fc-control treated animals (Fig. 3G, H), whereas colonic CD4^+^ Th1, Th17 or T regulatory cell subsets remained unaffected (Fig. 3I, J and data not shown). Since inflammation is driven by T cells in the colon, we next analyzed whether anti-IL12p40 treatment, which is known to reduce colonic T cells during colitis^28^, ameliorates colitis in our model. Consistent with previous reports, IL-12p40 blockade prevented weight loss (Fig. S8A), decreased inflammation score (Fig. S8B), the frequencies of inflammatory monocytes (Fig. S8C) and of colonic T cells (Fig. S8D, E). Altogether, this suggests that TNF produced by T cells drives inflammation during colitis.

TNF is known to induce the expression of multiple chemokines that recruit inflammatory myeloid cells to the sites of inflammation^5^. Thus, we next analyzed chemokine production in ex vivo cultured colonic explants from mice treated for two weeks with either anti-TNF or Fc controls (Fig. 3K–P). We detected a significant decrease in CCL3, CCL4, CXCL2 and CCL5, but not in CCL2 or CCL7 levels (Fig. 3K–P). Altogether, our results suggest that T cell-derived TNF regulates chemokine expression in the colon and perpetuates intestinal inflammation via recruitment of myeloid cells.

### T cell-derived TNF inhibits epithelial cell hyperplasia and restitution during established colitis

**Fig. 4:**
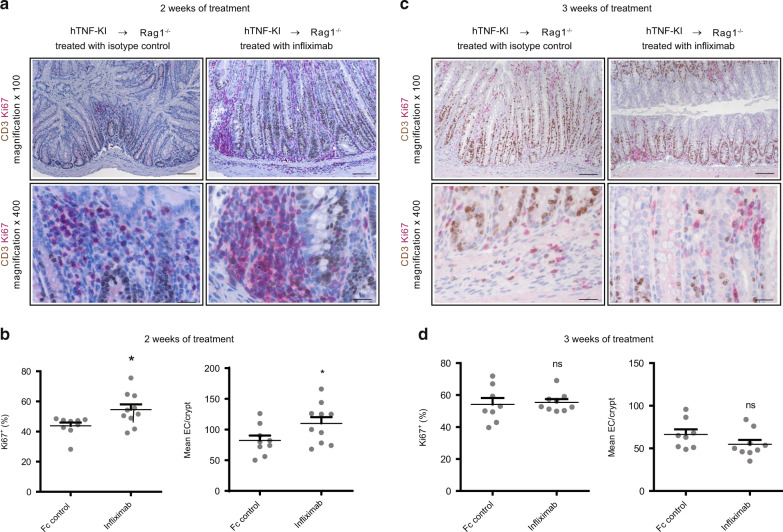
T cell-derived TNF blockade induces epithelial cell hyperplasia and restitution. Naive hTNF-KI T cells were transferred to Rag1^−/−^ recipients, anti-TNF (infliximab; 10 mg/kg; i.p. twice per week) was administered once mice lost 5% of their initial weight. **A** Representative images of colon tissue sections stained immunohistochemically for proliferation (Ki67, brown) and for CD3 expression (red) depict increased proliferation of colonic epithelial cells after 2 weeks of anti-TNF therapy. Scale bar is equal to 100 µm in x100 magnification and 25 µm in x400 magnification. **B** Increased frequencies of Ki67-positive epithelial cells and mean numbers of epithelial cells per crypt after 2 weeks of anti-TNF therapy as described in **A**. **C** Representative images of colon tissue sections stained immunohistochemically for proliferation (Ki67, red) and for CD3 expression (brown) show proliferation of colonic epithelial cells after 3 weeks of anti-TNF therapy. Scale bar is equal to 100 µm in x100 magnification and 25 µm in x400 magnification. **D** Frequencies of Ki67-positive epithelial cells and mean numbers of epithelial cells per crypt after 3 weeks of anti-TNF therapy as described in **C**. All data are representative of two independent experiments. Data represent mean values ± SEM. *p < 0.05, **p < 0.01, ***p < 0.001, as calculated by Student’s t-test; ns not significant.

**Fig. 5:**
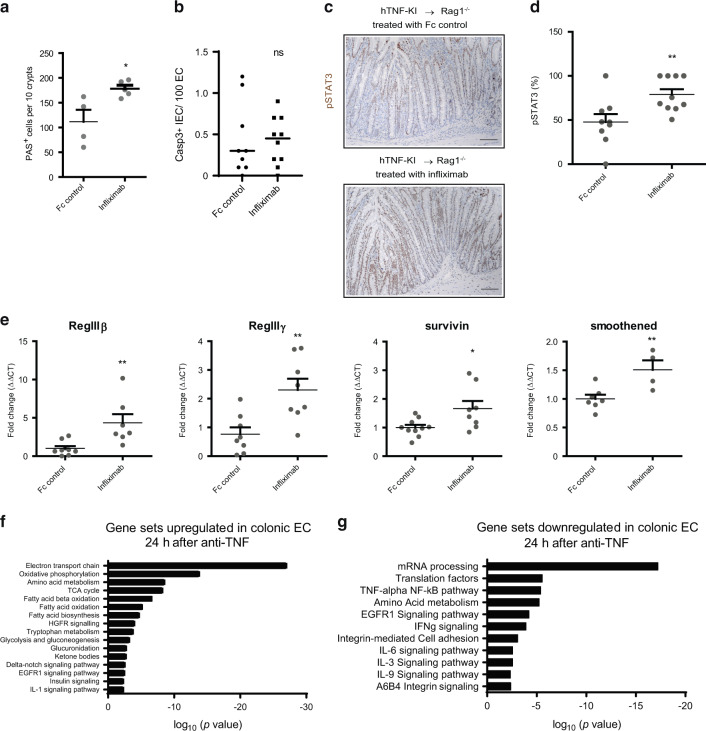
T cell-derived TNF represses STAT3 signalling in epithelial cells during colitis. **A** Numbers of goblet cells in the colon as revealed by PAS staining after 2 weeks of anti-TNF therapy. Data are representative of two independent experiments. **B** Numbers of apoptotic EC (Casp3 + ) per 100 EC in the colon after 2 weeks of anti-TNF therapy. Data collected using two independent experiments are shown. **C**, **D**. Representative images of colon sections stained immunohistochemically for pSTAT3 (brown) show increased pSTAT3^+^ epithelial cells upon anti-TNF therapy. **B**–**D** Data collected using two independent experiments are shown. Naive hTNF-KI T cells were transferred to Rag1^−/−^ recipients, anti-TNF (infliximab; 10 mg/kg; i.p. twice per week) was administered once mice lost 5% of their initial weight. Scale bar is equal to 100 µm. **E** Expression of STAT3 dependent genes (*Reg3β*, *Reg3γ*, *survivin*, *smoothened*) in the colon after 2 weeks of anti-TNF therapy. Data are representative of two independent experiments. **F** Gene sets upregulated in colonic EC (sorted live EpCAM1+ cells) 24 h after infliximab treatment, when compared to Fc-control treated group. **G** Gene sets downregulated in colonic EC (sorted live EpCAM1^+^ cells) 24 h after infliximab treatment, when compared to Fc-control treated group. **F**, **G** Data are from one experiment using epithelial cells from individual mice. Data represent mean values ± SEM. *p < 0.05, **p < 0.01, ***p < 0.001, as calculated by Student’s t-test; ns not significant.

In order to evaluate whether disease amelioration is accompanied by tissue repair as seen in IBD patients treated with anti-TNF agents, we analyzed the proliferation of epithelial cells upon T-TNF blockade. Two weeks of Infliximab administration led to epithelial hyperplasia, as indicated by increased Ki67-positive epithelial cells (Fig. 4A, B). Colons from mice receiving anti-TNF treatment for three weeks exhibited neither increased IECS proliferation, nor crypt elongation (Fig. 4C, D), suggesting that at this time point epithelial tissue restitution has ceased. Goblet cell numbers, as revealed by PAS staining, were increased upon anti-TNF therapy (Fig. 5A), while EC apoptosis was not affected (Fig. 5B), suggesting the restoration of epithelial layer functions upon anti-TNF therapy, as observed in IBD patients^29^. Altogether, these data indicated the restoration of the epithelial barrier during anti-TNF therapy.

STAT3 is involved in one of the crucial signaling pathways for epithelial cell repair and restitution of goblet cells during colitis^30,31^. Therefore, we next investigated whether increased IECs proliferation is associated with pSTAT3 expression in epithelial cells. Indeed, we observed that blockade of T-TNF resulted in increased pSTAT3 expression in IECs (Fig. 5C, D). Moreover, expression of several STAT3-dependent genes^30^, like those encoding antimicrobial peptides (*Reg3β*, *Reg3γ*) and molecules involved in tissue repair (*survivin*, *smoothened*) were significantly upregulated in the colon of infliximab-treated mice (Fig. 5E). Recently, TNF was proposed to contribute to mucosal repair via WNT signaling^32^. However, in our model we did not observe any difference in the expression of genes associated with WNT signaling upon T-TNF blockade for two weeks (Fig. S9 and Table S1). To gain further insight into early transcriptional changes in EC after anti-TNF, we sorted colonic epithelial cells one day after anti-TNF therapy (Fig. 5F, G and Table S1). Notably, TNF blockade upregulated genes associated with oxidative phosphorylation, TCA cycle and fatty acid β-oxidation, while inhibiting several proinflammatory pathways including IFNγ, IL-6 and IL-9 (Fig. 5F, G). Also here, genes associated with WNT signaling were not affected (Table S1).

Altogether, these data indicate that tissue repair after T-TNF blockade in established colitis is associated with rapid changes in the transcriptional program of epithelial cells.

### T cell-derived TNF interferes with epithelial cell proliferation via control of IL-22BP expression during established colitis

**Fig. 6:**
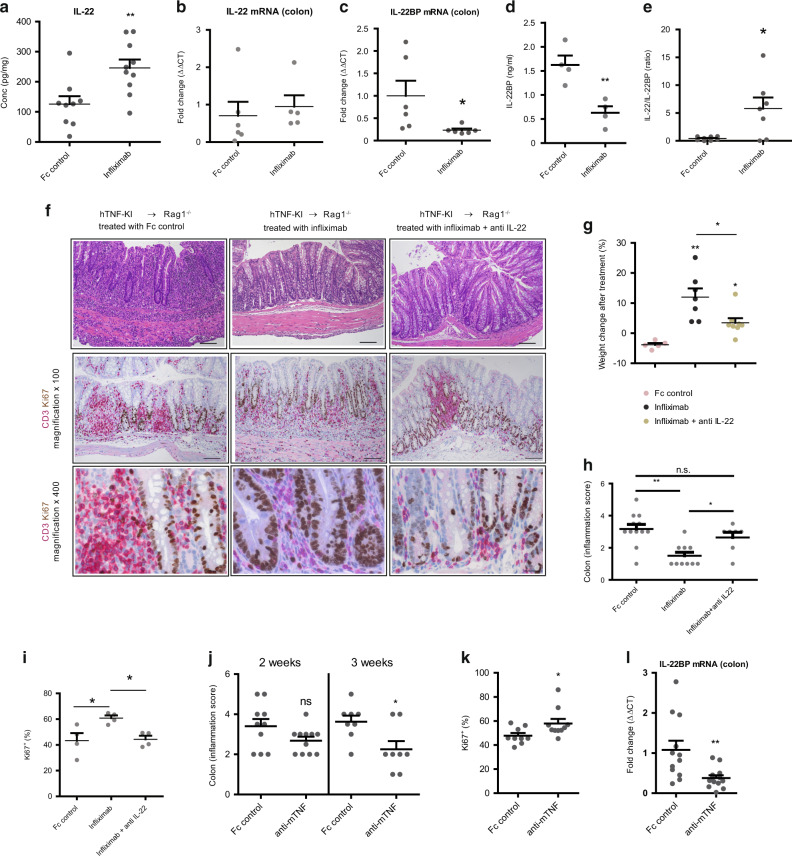
T cell-derived TNF modulates levels of IL-22 in vivo via control of IL-22BP expression during established colitis. **A** Levels of IL-22 in ex vivo colonic explants from mice treated with Fc control or infliximab for 2 weeks. **B** IL-22 mRNA levels in the colons of mice treated with Fc control or infliximab for 2 weeks. **C** IL-22BP mRNA levels in the colons of mice treated with Fc control or infliximab for 2 weeks. **D** Serum IL-22BP concentration in sera, **E** Ratio of IL-22 and IL-22BP mRNA levels in the colons of mice treated with Fc control or infliximab for 2 weeks. Naive hTNF-KI T cells were transferred to Rag1^−/−^ recipients, anti-TNF (infliximab; 10 mg/kg; i.p. twice per week) was administered once mice lost 5% of their initial weight. **F** Representative images of colon sections stained with Hematoxylin/Eosin and immunohistochemically stained for Ki67 (brown) and CD3 (red), **G** weight changes, **H** corresponding inflammation scores, **I** percentage of proliferating colonic epithelial cells after treatment of colitic mice with either Fc control, infliximab, anti-IL-22 or infliximab/anti-IL-22 (Fc control and infliximab - 10 mg/kg; anti-IL-22 - 10 mcg/mouse; i.p, twice per week) for 2 weeks. Naive hTNF-KI T cells were transferred to Rag1^−/−^ recipients, antibodies were administered once mice had lost 5% of their initial weight. Scale bar is equal to 100 µm in x100 magnification and 25 µm in x400 magnification. **J** Colonic inflammation score at various time points after start of anti-mTNF treatment. Percentage of proliferating colonic epithelial cells **K**, IL-22BP mRNA levels **L** in the colons of mice treated with Fc control or anti-mTNF for 2 weeks. All data are representative of two independent experiments. Data represent mean values ± SEM. *p < 0.05, **p < 0.01,***p < 0.001, as calculated by Student’s t-test; ns not significant.

To identify factors, which induce epithelial cell proliferation, we quantified the expression of several cytokines in the supernatants from ex vivo colonic explants. Strikingly, we observed that IL-22 expression was significantly upregulated after two weeks of T-TNF blockade, whereas the expression of other proinflammatory cytokines, like IL-6, IL-1β and IL10, remained unaffected (Fig. 6A and S10, S11). These results suggest that upon T-TNF blockade, IL-22 drives intestinal tissue repair. However, colonic *IL-22* mRNA levels were not significantly different between the groups (Fig. 6B), indicating that IL-22 function is regulated at posttranscriptional or at bioavailability level. Thus, we next analyzed the expression levels of IL-22BP upon T-TNF blockade and found that both colonic mRNA and serum protein levels were significantly downregulated compared to Fc control-treated mice (Fig. 6C, D), resulting in a significant increase of the IL-22/IL-22BP ratio in the colon (Fig. 6E).

To directly address whether IL-22 drives colonic epithelial cell proliferation and subsequent repair during colitis in vivo, we concomitantly neutralized IL-22 by blocking antibodies together with anti-TNF treatment (Fig. 6F). In this experiment, IL-22 contributed to the restoration of weight upon anti-TNF therapy (Fig. 6G), and subsequent reduction of inflammation (Fig. 6F, H). Strikingly, IL-22 ablation completely abolished epithelial tissue repair observed upon anti-TNF treatment as indicated by proliferating IECs (Fig. 6I) and *Reg3γ* induction (data not shown). Notably, in this set of experiments, Infliximab-mediated recovery occurred faster, which was reflected in rapid, extensive weight gain (Fig. 6G), and resulted in subsequent decrease in inflammation score two weeks after the initiation of anti-TNF therapy (Fig. 6H).

Data presented so far revealed that T cell-derived TNF controls IL-22 mediated epithelial restitution during colitis in the humanized TNF mouse model. Next, we addressed whether anti-mouse TNF blockade during colitis also affected IL-22BP expression. Consistent with TNF blockade in the humanized TNF model, mouse TNF blockade during colitis that was induced by transfer of WT T cells into Rag1^−/−^ recipients (i) reduced inflammation at 3 weeks after initiation of therapy (Fig. 6J), (ii) induced proliferation of epithelial cells (Fig. 6K) and (iii) inhibited IL-22BP production (Fig. 6L). Thus, TNF-mediated control of IL-22BP production occurs both in murine and humanized TNF colitis models.

IL-22 is known to be regulated by IL-18 in the gut during inflammation^33–35^ and anti-TNF therapy upregulated IL-18 expression (Table S1). Thus, we next assessed whether IL-18 directly contributes to colitis recovery induced by infliximab. However, concomitant ablation of IL-18 and T-TNF did not influence recovery of colitis (Fig. S12), suggesting that IL-18 is dispensable in this experimental setting.

### TNF regulates IL-22BP expression by dendritic cells

**Fig. 7:**
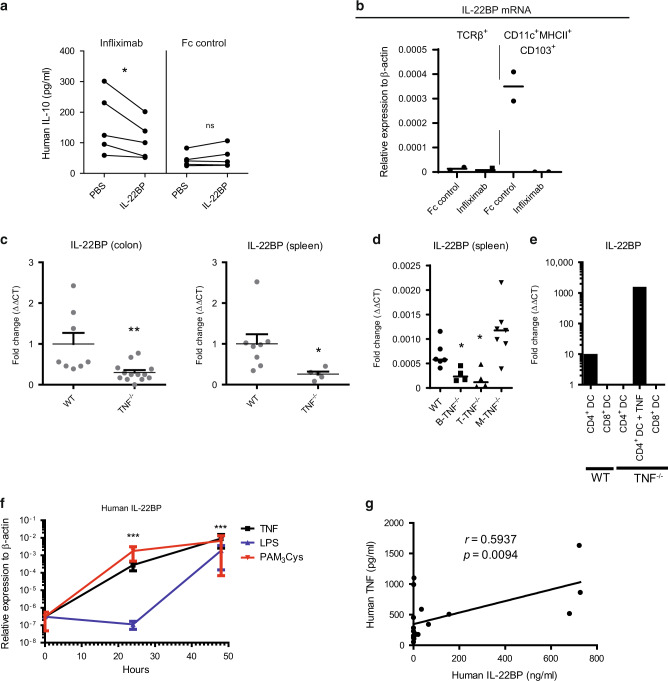
TNF controls IL-22BP expression in DCs. **A** TNF blockade increases levels of bioactive colonic IL-22 that can be inhibited by recombinant IL-22BP. Supernatants derived from colonic explants of Rag1^−/−^ mice which received naive hTNF-KI T cells and treated for 2 weeks with anti-TNF, were added to human colonic Colo205 cell line with or without rmIL-22BP (1,25 mg/ml). Production of human IL-10 as an indicator of bioactive IL-22 has been determined 48 h later. **B** Expression of IL-22BP mRNA in sorted CD45^+^CD11c^+^MHCII^+^CD103^+^ and CD45^+^TCRβ^+^ cells from the colon of mice treated with Fc control or infliximab for 2 weeks. Naive hTNF-KI T cells were transferred to Rag1^−/−^ recipients, anti-TNF (infliximab; 10 mg/kg; i.p. twice per week) was administered once mice had lost 5% of their initial weight. **C** Expression of IL-22BP in colon and spleen from naive WT and TNF^−/−^ mice. **D** Expression of IL-22BP in spleen from naive WT, T-TNF^−/−^, B-TNF^−/−^, and M-TNF^−/−^ mice. **E** Expression of IL-22BP in sorted CD4^+^CD11c^+^MHCII^+^ or CD8^+^CD11c^+^MHCII^+^ cells isolated from spleen of naïve WT and TNF^−/−^ mice. CD4^+^CD11c^+^MHCII^+^ cells from TNF^−/−^ mice were incubated with rmTNF (100 ng/ml; 4 h). **F** Levels of human IL-22BP in human monocyte-derived DCs (moDCs) stimulated with TNF (100 ng/ml), LPS (100 ng/ml), PAM_3_Cys (5 mcg/ml) for indicated times. **G** Correlation between hTNF and hIL-22BP levels in sera of IBD patients. All mouse data are representative of two independent experiments. Data represent mean values ± SEM. *p < 0.05, **p < 0.01, ***p < 0.001, as calculated by Student’s t-test; ns not significant. The Pearson correlation was used for correlative analyses. The significance level was set to *p* ≤ 0.05.

IL-22 protein levels were significantly upregulated two weeks after T-TNF blockade in colonic explants. Therefore, we next determined whether such an increase in protein levels would also result in enhanced IL-22 bioactivity (Fig. 7A). Indeed, supernatants of colonic explants from infliximab-treated animals induced higher IL-10 production by Colo205 cells as compared to Fc control treated mice. Notably, addition of recombinant IL-22BP protein reduced this effect (Fig. 7A). Thus, TNF likely reduces IL-22 bioactivity in vivo during colitis

IL-22BP production has been reported by many cell types^18–20,36^. Thus, we next sorted various cell subsets from the colons of anti-TNF or Fc- control treated Rag1^−/−^ mice, which were reconstituted with naive hTNF-KI T cells. IL-22BP mRNA expression analysis clearly demonstrated that TNF blockade abrogated IL-22BP production by intestinal dendritic cells (DCs), while its expression by T cells remained unaffected and was much lower than by DCs (Fig. 7B). IL-22BP expression was also reduced in spleen and colon of naive TNF-deficient mice (Fig. 7C). We next determined the cellular source of TNF responsible for IL-22BP production in the spleen and found that ablation of TNF in either B or T lymphocytes reduced splenic IL-22BP expression (Fig. 7D), indicating that multiple cellular sources may contribute to local IL-22BP production.

To assess whether TNF directly regulates IL-22BP expression, we sorted splenic CD4^+^ DCs and stimulated them with TNF. Firstly, we noted the lack of IL-22BP expression in splenic CD4^+^ DCs from *Tnf*-deficient mice (Fig. 7E). Next, we found that short-term TNF stimulation of TNF^−/−^ CD4^+^ DCs induced IL-22BP expression, implicating direct regulation by TNF of IL-22BP expression (Fig. 7E).

Human IL-22BP (hIL-22BP) can be induced by various TLR agonists, as well as by retinoic acid, in human monocyte-derived DCs (hmoDCs)^37^. Interestingly, we have observed that human TNF also significantly upregulated IL-22BP mRNA expression in hmoDCs as compared to unstimulated cells (Fig. 7F). Since TNF blockade affected not only colonic IL-22BP expression, but also its serum levels in the humanized mouse, TNF-dependent colitis model, we next quantified human IL22-BP in IBD patients. Also, here correlation between hTNF and hIL-22BP serum levels from IBD patients was observed (Fig. 7G). Of note, neither levels of hIL-22BP nor hTNF correlated with clinical score of IBD patients (Fig. S13).

Overall, our data indicate that TNF controls IL-22BP expression by DCs both in mice and humans.

### TNF produced by epithelial cells controls colonic expression of IL-22BP

**Fig. 8:**
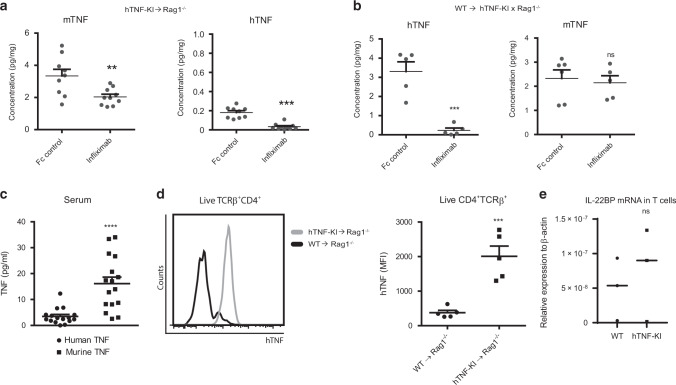
Contribution of individual cellular sources of TNF to epithelial cell proliferation and IL-22BP expression in the colon. **A** Levels of hTNF and mTNF in ex vivo colonic explants from Rag1^−/−^ mice following transfer of hTNF-KI T cells into Rag1^−/−^ mice and treated with Fc control or infliximab for 2 weeks. **B** Levels of hTNF and mTNF in ex vivo colonic explants from hTNF-KIxRag1^−/−^ mice that received T cells isolated from WT mice and were treated with Fc control or infliximab for 2 weeks. **C** hTNF and mTNF levels in sera of colitic Rag1^−/−^ mice transferred with hTNF-KI naive T cells before anti-TNF therapy. **D** Analysis of hTNF expression on the surface of blood T cells. Rag1^−/−^ mice were reconstituted with naive T cells from WT or hTNF-KI mice. Blood cells were analysed once mice had lost 5% of their initial weight. **E** Expression of IL-22BP in CD4 T cells isolated from WT and hTNF KI spleen. All data are representative of two independent experiments. Data represent mean values ± SEM. *p < 0.05, **p < 0.01, ***p < 0.001, as calculated by Student’s t-test; ns not significant.

To further dissect the mechanism of how TNF controls IL-22BP expression during colitis, we quantified human and murine TNF protein levels in ex vivo colonic explants upon TNF ablation. Blockade of T-TNF by Infliximab not only abrogated the production of hTNF protein, but also significantly decreased the production of mTNF by other cells (Fig. 8A). On the other hand, ablation of non-T cell derived TNF during established disease markedly reduced hTNF levels, leaving mTNF production unaffected (Fig. 8B). Interestingly, despite the lack of clinical improvement of colitis by blocking of TNF from “non-T cells”, levels of IL-22BP were reduced upon infliximab administration (Fig. S11L), indicating that “non-T” cell derived TNF may also regulate IL-22BP production. Thus, this suggested that both T cell-derived and “non-T”-derived TNF may control colonic IL-22BP production via distinct mechanisms.

Further analysis of human and murine TNF expression in sera of Rag1^−/−^ recipient mice of hTNF-KI T cells revealed significantly higher soluble murine TNF production as compared with soluble hTNF (Fig. 8C), suggesting that TNF by T cells is mostly produced in membrane-bound form during colitis. Consistent with this, blood T cells during colitis expressed TNF on their surface as detected by flow cytometry (Fig. 8D), but did not produce increased IL-22BP levels when compared to WT controls (Fig. 8E).

**Fig. 9:**
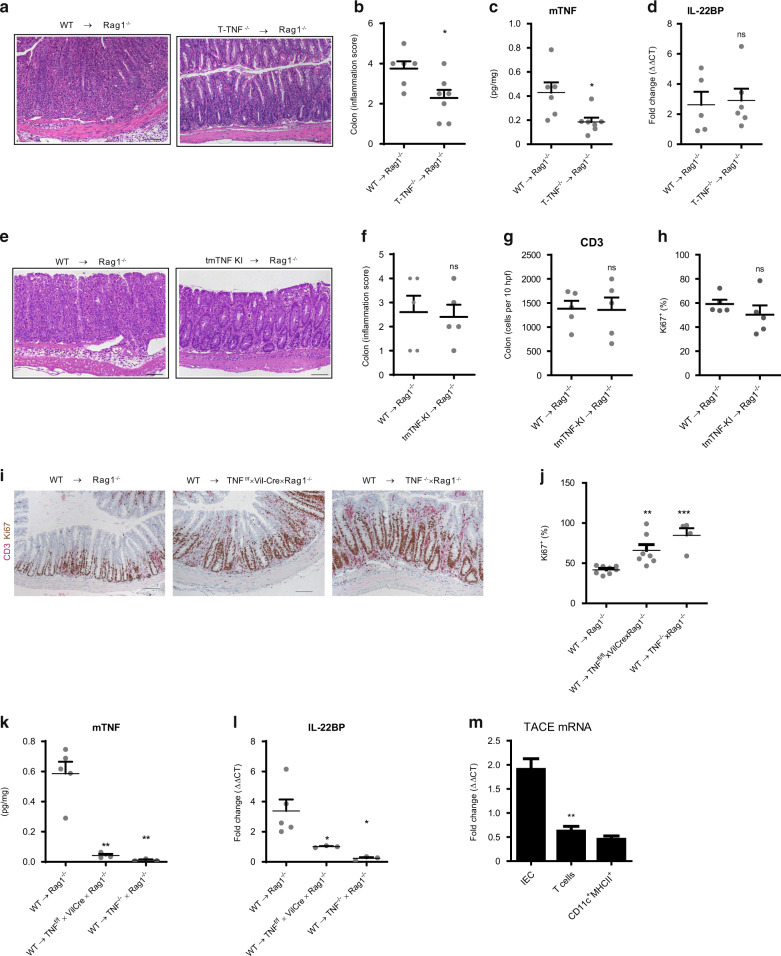
Contribution of individual cellular sources of TNF to epithelial cell proliferation and IL-22BP expression in the colon. Representative images of tissue sections stained with Haematoxylin/Eosin **A** (Scale bar is equal to 100 µm), colitis inflammation score **B**, mTNF levels in colonic explants **C** and expression of IL-22BP mRNA in colon **D** of Rag1^−/−^ mice reconstituted with naive T cells from WT or T-TNF^−/−^ mice. Representative images of tissue sections stained with Haematoxylin/Eosin **E** (Scale bar is equal to 100 µm), colitis inflammation score **F**, number of CD3 positive cells per 10 hpf in the colon **G** and frequency of proliferating epithelial cells in the colon **H** of Rag1^−/−^ mice reconstituted with naive T cells from WT or tmTNF KI mice. Representative images of colon sections stained for expression of CD3 (red) and Ki67 (brown) **I**, mean numbers of proliferating epithelial cells (Ki67 staining) **J**, mTNF levels in colonic explants **K**, IL-22BP mRNA levels in colonic tissue (**L**) of Rag1^−/−^, TNF^−/−^×Rag1^−/−^ and TNF^f/f^×Vil-Cre×Rag1^−/−^ mice reconstituted with naive T cells from WT donors. TACE mRNA expression levels in various cell subsets (IECs: CD45^-^ cells isolated from intraepithelial layer; T cells –CD45^+^TCRβ^+^CD4^+^ sorted from LP; CD11c^+^MHCII^+^ cells were sorted from LP) from Rag1^−/−^ reconstituted with WT naive T cells. All data are representative of two independent experiments. Data represent mean values ± SEM. *p < 0.05, **p < 0.01, ***p < 0.001, as calculated by Student’s t-test; ns, not significant.

To further dissect the role of soluble versus transmembrane TNF produced by T cells, we transferred naive T cells from WT, T-TNF^−/−^ or tmTNF-KI (mice expressing only membrane-bound TNF) mice into Rag1^−/−^ recipients (Fig. 9A–H). Mice which received TNF^−/−^ T cells lost less weight and exhibited a diminished inflammatory score (Fig. 9A, B). Ablation of TNF in T cells at the onset of disease resulted in mild reduction of TNF levels in the colon (Fig. 9C) and did not significantly affect IL-22BP expression levels (Fig. 9D). Consistent with this, transfer of T cells expressing non-cleavable transmembrane TNF (tmTNF-KI) induced fully blown colitis as severe as Rag1^−/−^ recipients of WT T cells (Fig. 9E, F, G, H). In sum, we conclude that TNF produced by T cells during colitis acts as membrane-bound molecule to promote inflammation.

Next, we determined the contribution of non-T cell derived TNF in IL-22BP mediated epithelial proliferation. To this end, we transferred sorted naive T cells into either Rag1^−/−^ TNF^f/f^ x Vil-Cre×Rag1^−/−^ or TNF^−/−^×Rag1^−/−^ recipients. Notably, colitis induced by transfer of WT T cells into TNF^f/f^ x Vil-Cre×Rag1^−/−^ and TNF^−/−^×Rag1^−/−^ recipients was accompanied by increased proliferation of epithelial cells (Fig. 9I, J), in contrast to Rag1^−/−^ mice reconstituted with WT T cells. This increase in proliferation was associated with significantly reduced TNF production in the colon and with decreased IL-22BP expression (Fig. 9K, L). Given that measurements of colonic explants reflects the abundance of soluble cytokines in the medium, our data further indicate that soluble TNF during colitis is likely be produced by non-T cells.

Both myeloid and epithelial cells produced TNF that contributes to colitis^38,39^. Thus, we next genetically depleted TNF in myeloid cells (M-TNF^−/−^× Rag1^−/−^; TNF^f/f^×Mlys-Cre×Rag1^−/−^) and in epithelial cells (EC-TNF^−/−^×Rag1^−/−^; TNF^f/f^×Vil-Cre×Rag1^−/−^) in Rag1^−/−^ mice and induced colitis by transfer of naive T cells from WT mice. Strikingly, these experiments revealed that TNF produced by epithelial cells can control epithelial cell repair, colonic TNF production and IL-22BP colonic levels (Fig. 9I–L), while myeloid cell derived TNF contributed to severity of colitis without influencing IL-22 levels in the colon (Fig. S14). Since soluble TNF is produced upon cleavage of tmTNF by metalloproteases, such as ADAM17 (TACE), we next measured TACE expression in various cell subsets isolated from the colon. Interestingly, we observed high expression of TACE in epithelial cells, but not in DCs nor in T cells (Fig. 9M).

Our results indicate that membrane-bound T-TNF is involved in perpetuated intestinal inflammation that also results in increased IECs-derived TNF. At the same time, soluble TNF from epithelial cells controls IECs proliferation via IL-22BP induction and, thereby, regulates tissue repair.

### Contribution of TNFR2 signaling to the recovery from colitis upon anti-TNF therapy

**Fig. 10:**
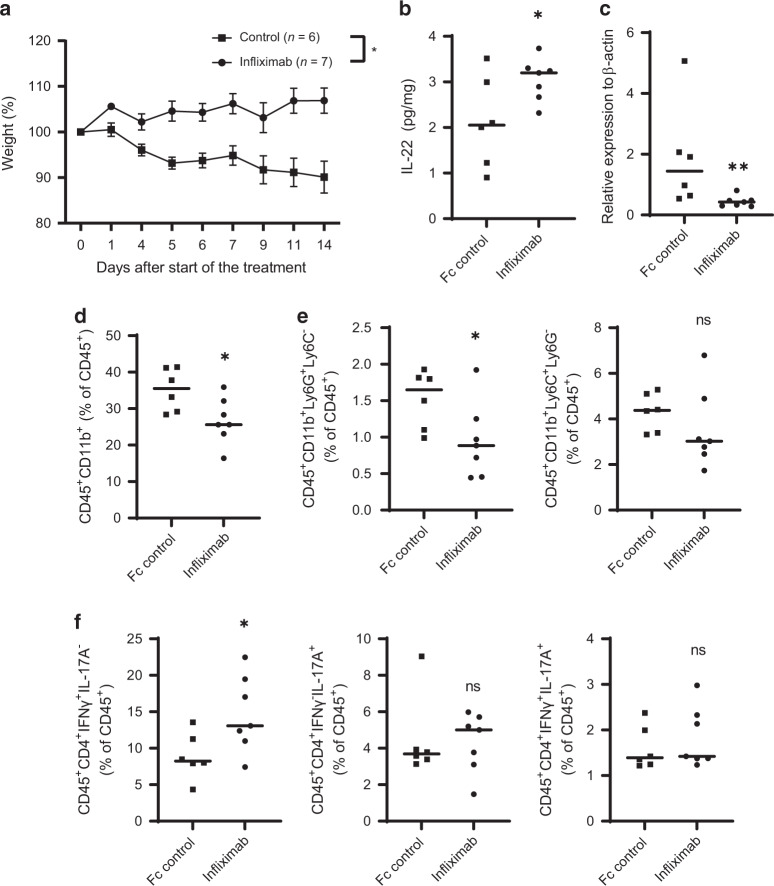
T cell-derived TNF is pathogenic during established colitis in hTNFRp75-KIxRag1^−/−^ mice. **A** Weight of hTNFRp75‐KIxRag1^−/−^ mice reconstituted with naive T cells from hTNF‐KI mice and treated with Infliximab or Fc control (10 mg/kg) twice per week. IL-22 levels in colonic explants **B** and expression of IL-22BP mRNA in colon **C** of hTNFRp75‐KIxRag1^−/−^ mice reconstituted with naive T cells from hTNF-KI mice and treated with-infliximab for 2 weeks. Frequencies of CD11b^+^ (CD45^+^CD11b^+^) **D** of granulocytes (CD45^+^CD11b^+^Ly6G^+^Ly6C^+^) and inflammatory monocytes (CD45^+^CD11b^+^Ly6G^-^Ly6C^+^) **E**, Th1 (CD45^+^CD4^+^TCRβ^+^IFNγ^+^) **F**, Th17 (CD45^+^CD4^+^TCRβ^+^IL-17A^+^) **G** and Th1/17 (CD45^+^CD4^+^TCRβ^+^IL-17A^+^IFNγ^+^) **H** cells 2 weeks after infliximab or Fc control therapy. All data are representative of two independent experiments. Data represent mean values ± SEM. *p < 0.05, **p < 0.01, ***p < 0.001, as calculated by Student’s t-test; ns not significant.

Human TNF has a weak binding affinity to murine TNFR2^40^. Thus, next we aimed to evaluate the impact of TNFR2 signaling on anti-TNF induced recovery during established colitis. To this end, we induced colitis by transfer of naive hTNF-KI T cells into Rag1^−/−^ recipients expressing human TNFR2 as a KI (hTNFRp75-KIxRag1^−/−^ mice)^41^ and treated mice with infliximab or respective control (Fig.10). Mice treated with infliximab rapidly gained weight when compared with Fc-treated control animals (Fig. 10A), indicating that T-TNF is also pathogenic in this setting. Moreover, the infliximab-treated group exhibited increased IL-22 protein levels (Fig. 10B), while IL-22BP mRNA levels were inhibited by T-TNF blockade (Fig. 10C). Further, anti-TNF therapy reduced frequencies of myeloid cells, increased numbers of Th1 CD4 T cells and was dispensable for Th17 and Th1/17 CD4 T cells (Fig. 10D–F). Altogether, this data suggest that humanization of TNFR2 signaling is dispensable for the recovery from colitis, regulation of IL-22/IL-22 BP expression and recruitment of myeloid cells.

## Discussion

Efficient treatment of inflammatory bowel disease (IBD) requires not only blockade of ongoing inflammation, but also complete restoration of tissue functions. Thus, restoration of epithelial cell functions during IBD is the major target for IBD treatment. IECs homeostasis is maintained by immune cells in the gut and by microbial composition^1,2,42^. Perturbations of either regulatory arm may result in impaired epithelial barrier functions leading to chronic intestinal inflammation characterized by increased expression of proinflammatory cytokines, such as TNF, IL-6 and IL-1β^5^. However, systemic blockade of only TNF in IBD patients may significantly ameliorate inflammation and induce tissue healing^5^, although the mechanisms of immune-mediated tissue repair in IBD remained elusive. In this study, we demonstrate that in IBD, TNF controls IL-22BP expression and thereby, through modulation of IL-22 activity, limits repair of the epithelial cell layer.

TNF is a pleiotropic cytokine that exerts both beneficial and deleterious functions during autoimmunity^43–45^. Our new data further detail the mechanism of how anti-TNF therapy perpetuates epithelial tissue repair via modulation of IL-22 bioactivity. A recent study suggested a promotion of intestinal epithelial tissue repair by TNF via induction of WNT signaling^32^. In that study increased proliferation of epithelial cells in anti-TNF refractory patients was interpreted as indication for TNF-promoted IECs proliferation. However, the patient cohort responding to anti-TNF therapy also showed mucosal tissue repair^3^ and no cases of epithelial barrier leakage upon anti-TNF therapy have been reported so far, further advocating that anti-TNF treatment does induce IECs repair when it is efficacious. Intriguingly, our study employing a small cohort of IBD patients revealed a correlation between human TNF in sera and IL-22BP. At the same time, these parameters did not correlate with disease severity. Importantly, we were not able to measure bioactive TNF by existing assays because sera from IBD patients induced TNF-independent cell death of both reporter cell lines tested. Altogether, our data revealed a strong correlation between serum protein levels of TNF and IL-22BP during IBD and further studies showing whether patients with high TNF and IL-22BP levels exhibit IECs repair upon anti-TNF therapy are required.

In order to combat invading pathogens, inflammation must be induced by immune protective mechanisms involving numerous cytokines, which drive host defense against pathogens. In such situations, all host resources should be directed towards the aim to eradicate the pathogen and induce the repair before resolution of inflammation may become deleterious^46^. One possible scenario is that the inflammatory processes may be directly coupled to the repair program in the tissue. In line with this, our data demonstrate that TNF interferes with IL-22 bioactivity through induction of IL-22BP implying that inflammatory processes may limit tissue repair. Exploiting TNF blockade in established chronic inflammatory disorders using a novel mouse model recapitulating human pathology, we demonstrate that TNF orchestrates two distinct pathways in perpetuating chronic intestinal inflammation: first, by chemokine-mediated recruitment of inflammatory myeloid cells and second, by interference with epithelial cell proliferation via control of IL-22 activity in the colon. Thus, our data indicate that tissue repair and inflammation in some chronic inflammatory models are tightly interconnected.

IL-22BP is produced by various cell types such as DCs, T cells, eosinophils, and epithelial cells^19,20,36^. Moreover, enhanced IL-22BP protein expression was recently reported in CD4 T cells and in DCs isolated from IBD patients^20^. Only T cell-derived IL-22BP expression appeared to be affected by anti-TNF therapy in one of these studies, although TNF did not induce IL-22BP in T cells directly^20^. Here we showed that TNF induces IL-22BP expression by DCs in vitro. Additionally, our data demonstrate that TNF blockade during colitis results in decreased IL-22BP production by intestinal DCs, but not by T cells, suggesting different modes of regulation of IL-22BP expression by these two cell types. Discrepancies between previous reports and this study could be explained by different sorting strategies: we sorted CD103^+^CD11c^+^MHCII^+^ cells, whereas previous works focused on CD11c^+^ cells only. Dissection of the mechanisms of transcriptional regulation of IL-22BP in distinct cell types is required to fully understand the contribution of each IL-22BP producing cell subset in modulating IL-22 bioactivity in vivo.

Further analysis of IL-22BP expression in naive animals showed that both T-cell and B-cell derived TNF contribute to control of IL-22BP expression in the spleen. T-TNF blockade during established colitis not only eliminated T-TNF, but also decreased levels of non-T cell-derived TNF possibly by limiting inflammation. Finally, genetic ablation of TNF in distinct cellular sources in this colitis model revealed that soluble TNF produced by IECs controls IL-22BP expression in the colon, while transmembrane T cell-derived TNF regulated inflammation and TNF production by IECs and, thereby, Il-22 bioactivity. Thus, TNF produced by distinct cellular sources controls IL-22BP expression in various tissues and this warrants the further dissection of contribution of its source in various disease conditions.

Of note, we have observed a discrepancy in genetic and pharmacological TNF inhibition on IL-22BP production: blockade of T cell-derived TNF during established colitis induced tissue repair via the IL-22/IL-22BP axis, while genetic depletion of TNF in T cells did not affect IL-22BP. Further experiments revealed that IECs restitution upon anti-TNF therapy is a tightly controlled, time-limited process, since it was evident at 2 weeks, but not at 3 weeks, after initiation of the treatment and, thus, it was not evident once TNF^−/−^ T cells were transferred into Rag1^−/−^ recipients. Further studies are needed to uncover the exact mechanism restoration of IECs homeostasis upon cessation of colonic inflammation.

TNF blockade possesses both regenerative and destructive functions depending on the context of the inflammatory reaction^47–49^. Chronic intestinal inflammation induced by TNF overproduction contributed to IECs apoptosis and development of ileitis^47^. On the other hand, TNF protects IECs in the acute gut ischemia-reperfusion model via NFkB activation^48^. Our results further highlight deleterious effects of TNF on epithelial cell homeostasis in a chronic model of intestinal inflammation. Subsequent in vitro studies revealed that a chronic, but not a short term, exposure of epithelial cells to TNF resulted in decreased cell proliferation and death^49^. Hence TNF may exert its pathogenic functions by interfering with the maintenance of the epithelial barrier integrity in chronic inflammation.

Chronically elevated TNF levels are associated with aging and aging-associated pathologies^50^ and IL-22 may ameliorate some of these disorders^51^. Thus, it appears plausible that TNF affects the development of aging-associated diseases via modulation of IL-22 bioactivity. This possible link has to be validated in future studies.

Anti-TNF therapy, although effective in many patients, is associated with unavoidable side effects, including exacerbations of preexisting skin diseases^52^. Of note, most inflammatory skin disorders are associated with IL-22 and IL-22 blockade is beneficial in these patients^16^. Our results provide explanation how anti-TNF treatment increases IL-22 bioavailability and, thus, exacerbates such diseases. Further studies in patients undergoing anti-TNF therapy who develop secondary autoimmune manifestations in the skin will be needed to address this.

## Materials and methods

### Study design

The objectives of the present study were to establish humanized TNF mouse models and harness them for in-depth analysis of biological effects of anti-TNF drugs and dissection of the mechanism of anti-TNF mediated epithelial cell restitution. Both males and females were used in all experiments. Animals were allocated to experimental groups based on their genotype; mice in one cage were assigned to various treatment regimens to reduce cage-to-cage effect. We did not use exclusion approaches for assignment of the animals to the experiments. For treatment with various agents, mice were blindly assigned to the respective group by an independent scientist. All injection and dissection experiments were conducted in a non-blinded fashion. The sample sizes were determined on the basis of previous experience. Blinding was used for all the mouse analysis, including histology. For each experiment, sample sizes reflect the number of independent biological replicates and are provided in figure legends. In general, sample size was chosen to use the least number of animals to achieve statistical significance according to 3 R regulations.

### Mice

Mice expressing human TNF (hTNF-KI mice) were generated in our laboratory (Fig. S1)^21,22^. Mice with ablation of TNF in T cells (T-TNF^−/−^ mice; TNF^flox/flox^×CD4-Cre; RRID:MGI:3527254), with ablation of TNF in myeloid cells (M-TNF^−/−^; TNF^flox/flox^×Mlys-Cre; RRID:MGI:3527247), with ablation of TNF in B cells (B-TNF^−/−^; TNF^flox/flox^ ×CD19-Cre; RRID:MGI:3527259), with ablation of TNF in epithelial cells (EC-TNF KO; TNF^flox/flox^ ×Villin-Cre), TNF KO (RRID:MGI:3582275), tmTNF KI^53^, hTNFRp75 KI^41^ and Rag1^−/−^ (RRID:MGI:3850561) mice used in this work were previously described^43,54,55^. Ablation of TNF in all cells, in myeloid cells and epithelial cells of Rag1^−/−^ mice was achieved by backcrossing TNF KO, M-TNF KO and EC-TNF KO on Rag1^−/−^ background. hTNF-KI and hTNFRp75 KI mice on Rag1^−/−^ background were bred for this study.

TNF^flox/flox^(TNF^<tm1SNed>^/TNF^<tm1SNed>^) mice were used as wild-type (WT) controls for all experiments performed with T-TNF KO mice and as donors of naive T cells when T-TNF KO T cell transfer was performed. C57BL/6 mice were purchased from Charles River Laboratories. All mice were kept under specific pathogen-free conditions in the animal facility of German Rheumatism Research Centre (DRFZ), Berlin, and Pushchino animal facility and were used for experiments at 6-12 weeks of age. Mice from various experimental groups were cohoused to exclude effects of microbiota variations on the observed phenotype. Mice in every cage were randomly assigned to various treatments to exclude cage effect. All animal procedures were performed in accordance with German and Russian regulations of animal protection.

#### IBD patient blood samples

Blood samples were obtained from IBD (both UC and CD) patients attending the outpatient clinic at Charité Campus Benjamin Franklin after informed consent. The study was approved by the local ethical committee (EA4/014/18). Clinical response was routinely assessed at week 6 after initiation of anti-TNF treatment (Infliximab) and defined as a decline in the Partial Mayo Score of at least 3 points for patients with ulcerative colitis and at least 3 points in the Harvey-Bradshaw Index for patients with Crohn´s disease, or by global physician assessment. Patients were classified as responders or non-responders based on their primary response to infliximab treatment. Clinical score had been determined at the time of blood collection.

### Isolation of CD4^+^CD45RB^hi^CD25^−^ T cells

Spleens and peripheral lymph nodes (LN) (superficial cervical LN, submandibular LN, axillary LN, accessory axillary LN, inguinal LN and mesenteric LN) were collected from donor mice. Cells were isolated by homogenizing spleens and LN via cell strainers (80 µm) (BD Falcon) in PBS/BSA under sterile conditions in a Laminar Flow Box (Thermo Fischer Scientific). Cells were pelleted by centrifugation (1500 rpm, 7 min) and the supernatant was discarded. For red blood cell depletion, cells were resuspended in ACK erythrocyte lysis buffer, incubated for 5 min at 4 °C and washed. Isolated cells were enriched for CD4^+^ T cells by magnetic-activated cell sorting (MACS) negative selection using anti-biotin microbeads (Miltenyi Biotec; Cat #: 130-097-046). Briefly, 5×10^8^-1×10^9^ cells were incubated with biotinylated anti-CD8, anti-CD25 and anti-B220 monoclonal antibodies for 15 min at 4 °C followed by streptavidin-conjugated magnetic beads (Miltenyi Biotec) in PBS/BSA. All washing steps were performed at 1500 rpm at 4 °C in PBS/BSA. Before MACS separation, cells were filtered through 30 µm mesh filters (Miltenyi Biotec). The Automacs program possel (positive selection) was used for separation.

Upon magnetic sorting, the negative fraction containing CD4^+^ T cells was incubated with CD4-PE-Cy7, CD45RB-PE, and CD25-APC antibodies, as well as propidium iodide (PI) to exclude dead cells. Cells were further sorted by fluorescence-activated cell sorting (FACS) on FACS Aria II (BD Biosciences). Anti-CD25 staining excluded CD25^+^ T cells, and the CD4^+^CD45RB^hi^ CD25^-^ population was sorted with a purity of >98%.

### Colitis induction and monitoring

Rag1^−/−^ animals were reconstituted by injection of 3 × 10^5^ sorted CD4^+^CD45RB^hi^CD25^-^ cells in 200 µl PBS per mouse. At the time of transfer, the recipient mice were weighed and subsequently monitored daily. Mice were sacrificed after 40–60 days post transfer. Treatment with anti-human TNF drugs or respective isotype control was performed once weight loss was >5% for twice a week for two or three weeks, as indicated, and then sacrificed. Tissue samples were removed for histology, cell isolation, preparation of colonic explants and RNA isolation.

### Treatment of colitic mice

Once mice had lost >5% of initial weight, they were treated with anti-human TNF antibody (Infliximab), anti-mouse IL-22 (R&D Systems; AF582) or human Fc-IgG1 isotype control (BioXCell; Cat # BE0096) in PBS. Anti-human TNF drugs were kindly provided by Dr. Eugen Feist (Charité, Universitätsmedizin Berlin). Antibodies were injected intraperitoneally (i.p.) in 100 µL PBS at a dose of 10 mg/kg body weight twice a week for two or three consecutive weeks. Anti-mouse IL-22 antibody was injected i.p. at a dose of 10 µg per mouse in 200 µL PBS. Mice were analyzed 3 days after the last injection. Anti-IL-12p40 antibody (clone C17.8; 10 mg/kg, i.p.; twice per week), anti-mTNF (BioXcell; clone XT3.11; 10 mg/kg, i.p.; twice per week) or anti-IL-18 (Bioxcell; clone YIGIF74-1G7; 10 mg/kg, i.p.; twice per week) or respective isotype controls were used for respective cytokine blockade.

### Organ isolation

Mice were sacrificed and their mesenteric LN (mLN) and colons were collected. LN cells were obtained as described earlier (Isolation of CD4^+^CD45RB^hi^CD25^-^ T cells). The colon was cleaned of mesenteric fat using forceps and the length from the cecum to rectum was determined with a ruler. The colon was subsequently divided into four equal pieces from proximal to distal part for cell isolation, ex vivo culture, histology and RNA isolation, respectively.

### Histological examination

Colon tissue samples were flushed with PBS and fixed in 4% formalin at 4 °C overnight. After fixation, tissue samples were dehydrated and immersed in xylene and embedded in paraffin. 1–2 μm paraffin sections were cut, dewaxed and stained histochemically with hematoxylin and eosin or immunohistochemically with the respective antibodies (pSTAT3 (Clone: EP2147Y; Abcam; Cat #: ab76315), CD3 (DAKO; Cat #: N1580), KI67 (clone: TEC3; DAKO; Cat #: M7249), Casp3 (Clone: Asp175; Cell Signaling; Cat #: 9664 S) and F4/80 (Clone: BM8; Thermo Fischer Scientific; Cat #: 14-4801), followed by Streptavidin-AP kit (DAKO; Cat #: K5005) and Envision PO kit (DAKO; Cat #: K5007). The sections were processed, stained and analyzed in a blinded fashion. Representative pictures were taken by a pathologist and were provided further to the scientist for decoding and illustrations of disease characteristics. The degree of colonic inflammation was scored using a previously described scoring system by Erben and colleagues^56^.

### Colonic explants

Colonic tissue from mice was thoroughly cleaned with cold PBS/BSA to remove excess mucus and feces. Samples were weighed and incubated in 1 mL of RPMI containing 10% FCS and penicillin/streptomycin (100U/ml) in a 48-well plate. The colonic explants were incubated for 24 h at 37 °C in 5% CO_2_. Medium was collected, centrifuged, pelleted cell debris discarded and the supernatant stored at −20 °C.

### Cell isolation

For isolation of lamina propria lymphocytes, colon samples were longitudinally cut, flushed with cold PBS/BSA and cut into small pieces. Pieces were transferred into RPMI containing 5% FCS and 5 mM EDTA and incubated for 20 min at 37 °C to remove epithelial cells and to isolate intraepithelial lymphocytes (IELs). Samples were vigorously shaken; supernatant was removed and replaced with fresh EDTA solution and the incubation was repeated. The IEL-containing supernatants were washed before further experimental procedures. Intestinal pieces were minced with scissors and scalpel and were transferred into fresh 50 mL Falcon tubes to be digested in RPMI containing 5% FCS and 1 mg/mL collagenase D (Roche; Cat #: 11 088 866 001) and 1 mg/mL dispase II (Sigma-Aldrich; Cat # D4693). After 20 min of digestion at 37 °C in a shaking incubator (200 rpm), samples were thoroughly vortexed and the cell-containing supernatant was collected and washed. Undigested tissue was incubated with fresh digestion mix for another 20 min in the shaking incubator. Samples from two digestion rounds were pooled and filtered through 80 µm filters (BD Falcon). After washing, cells were used for further experimental procedures.

Splenic DCs were isolated as follows: spleens were digested in RPMI-1640 containing 10% FCS, penicillin/streptomycin (100U/ml), 0,5 mg/ml of DNAse 1 (Sigma-Aldrich; Cat # DN25) and 0,8 mg/ml of Collagenase type IV (Sigma –Aldrich; Cat # C5138). Splenocytes were stained with antibodies against CD11c, MHCII, CD4 and CD8 (for details, see flow cytometry section), as well as propidium iodide (PI) to exclude dead cells. Cells were further sorted by fluorescence-activated cell sorting (FACS) on FACS Aria II (BD Biosciences). Resulting DC populations were sorted with a purity of >98%. Sorted cells were further activated by rmTNF (10 ng/ml; Myltenyi Biotec; Cat #: 130-101-688) and expression of IL-22BP was analysed.

### Isolation of colonic epithelial cells for RNA sequencing

Colon samples were longitudinally cut, flushed with cold PBS/BSA and cut into small pieces. Pieces were transferred into PBS with 5 mM EDTA, 1 mM DTT and incubated for 20 min at 37 °C to remove epithelial cells and to isolate IEL. Samples were vigorously shaken; supernatant was removed and replaced with fresh EDTA solution and the incubation was repeated. The IEL-containing supernatants were centrifuged and resuspended in PBS containing (0,05% of collagenase D (Roche), 0,005 % of DNAseI (Sigma-Aldrich) and 0,3 % of Dispase II (Roche) and were and incubated for 20 min at 37 °C. Cells were washed and used for further staining.

### Gene expression analysis

To assess transcriptome profiles of highly pure (>99%) live epithelial cells (EpCAM1-positive) total RNA was isolated using RNeasy Plus Micro kit (Qiagen) according to the manufacturer’s instructions. Illumina libraries were prepared using Smart-Seq v4 mRNA Ultra Low Input RNA Kit (Clontech) and Nextera XT DNA Sample Preparation Kit (Illumina), with up to 10 ng of purified cDNA, according to the manufacturer’s instructions. Libraries were paired-end sequenced (2 × 75 bp) on an Illumina NextSeq500 device. Raw sequences were mapped to mouse GRCm38/mm10 genome with TopHat2 in very sensitive settings for Bowtie2. Per gene counts were calculated using featureCounts and gene expression analyses were performed using DESeq2 1.18. Differential expression between Fc-treated and infliximab-treated ECs was regarded as significant when the adjusted p value was <0.05 and the fold change was >1.3. The resulting gene sets were further analysed for pathway enrichment by Enrichr^57^.

### Human monocyte-derived dendritic cells (hmoDCs) generation

Experiments with human blood material were approved by the Ethics Committee of the Charité - Universitätsmedizin Berlin, Germany [EA2/064/14]. The buffy coats were obtained from healthy donors through the blood bank of the German Red Cross (Deutsches Rotes Kreuz, DRK). Donors were kept anonymous.

Peripheral blood mononuclear cells (PBMCs) were isolated from buffy coats by density gradient centrifugation (Biocoll, Biochrom GmbH, DE). After thorough washing with phosphate buffer saline containing 2% EDTA and 1% human serum, monocytes were isolated as CD14^+^ cells from the PBMC fraction by positive magnetic bead isolation according to manufacturer’s instructions (Miltenyi Biotech, DE). To generate hmoDCs, 1.5 mln of monocytes were cultured in 6-well plates in RPMI-1640 (Gibco, IRL) supplemented with heat inactivated 10% (v/v) fetal calf serum (FCS) (Lonza Group, CH), 1% L-glutamine, 1% sodium pyruvate, 1% MEM non-essential amino acids, streptomycin/penicillin (100U/ml), 0.1% β- mercaptoethanol (β-ME) (Gibco, IRL), recombinant IL-4 (500 U/ml) and GM-CSF (800 U/ml; PeproTech, NJ, USA), at 37 °C and 5% CO_2_. Medium was changed after 2 days and mo-DCs were harvested after 5 days. Cells were then stimulated with rhTNF (100 ng/ml), LPS (Sigma-Aldrich; Cat # L2630; 100 ng/ml) and Pam_3_Cys (Invivogen; Cat# tlrl-pms; 5 mcg/ml) for various times. Cells were then lysed using TRI reagent, mRNA was isolated and gene expression was measured by real-time PCR.

### Cell activation

For analysis of cytokine production, 1×10^5^-1×10^6^ cells were incubated in IMDM medium containing 10% FCS and penicillin/streptomycin (100U/ml) in a 96-well plate in the presence of PMA (5 ng/mL) (Sigma-Aldrich; Cat #: P8139) and Ionomycin (500 ng/mL) (Sigma-Aldrich; Cat #: I0634) for 4 h at 37 °C. For intracellular accumulation of cytokines, 10 µg/mL Brefeldin A (Sigma-Aldrich; Cat #: B7651) was added for the time of stimulation.

### IL-22 bioactivity assay

Colo205 cell line (provided by Dr. Verena Moos, Charite, Berlin; RRID: CVCL_0218) was used to estimate IL-22 bioactivity. Briefly, 40,000 cells/well were seeded in a 96-well plate. The supernatants from colonic explants with or without addition of rmIL-22BP (RnDSystems; Cat #: 2376-BP-025) or anti-IL-22 (RnDSystems; Cat # MAB5821) were added to the culture for 48 h. Afterwards, supernatants were collected and the amount of IL-10 produced was measured using a human IL-10 Ready-Set-Go! ELISA Kit (Thermo Fischer Scientific; Cat #: 88-7106-22).

### Apoptosis induction by infliximab in mouse splenocytes

Splenocytes were isolated as described in (Isolation of CD4^+^CD45RB^hi^CD25^−^ T cells).

Cells (5 × 10^5^ cells/well) were plated in RPMI‐1640 complete media (10% FCS, 2 mM L glutamine, penicillin/streptomycin (100U/ml). Cells were stimulated with LPS (Sigma-Aldrich; Cat #: L2880; 50 μg/ml), with or without infliximab or respective Fc control (final concentration is 20 μg/ml). Cells were incubated at 37 °C for 24 h and were stained with Propidium iodide and Annexin V-Cy5, according to the manufacturer’s instructions (Thermofisher Scientific).

### Flow cytometry

In order to analyze various cellular subsets by flow cytometry, isolated cells were stained according to the guidelines^58^. Up to 10^7^ viable cells were resuspended in 100 µL PBS/BSA and antibodies for surface molecules (Table 1) were added in the optimal concentrations (as supplied and evaluated by titration). Samples were incubated for 15 min at 4 °C in the dark. Subsequently, cells were washed with PBS/BSA, centrifuged and resuspended in an appropriate volume.

Secondary staining of biotinylated antibodies was performed with Streptavidin-PE (Thermo Fischer Scientific). To distinguish viable and dead cells, live/dead fixable dead cell stain (Thermo Fischer Scientific), DAPI (Thermo Fischer Scientific; Cat #: D1306) or propidium iodide (Sigma-Aldrich; Cat #: P4864) was used.

For intracellular staining of cytokines and transcription factors, cells were fixed and permeabilised using the Foxp3 intracellular staining kit (Thermo Fischer Scientific; Cat #: 00-5523-00). For this, cells were incubated with freshly prepared Fix/Perm buffer (Diluent/Concentrate 3:1) for 20 mins at 4 °C, followed by washing with Permeabilisation buffer (diluted 1:10 in H_2_O), centrifugation and incubation with respective antibodies (Table 2) for 15 min at 4 °C.

**Table 2:** Monoclonal antibodies used for immunofluorescence staining (intracellular).

Antibody	Fluorochrome	Clone	Company	Identifier
IFNγ	BV 421	XMG1.2	BioLegend	Cat # 505829
IL-17A	APC	eBio17B7	Thermo Fischer Scientific	Cat # 17-7177-81
IL-22	PerCP eFluor 710	IL22JOP	Thermo Fischer Scientific	Cat # 46-7222-82
Rorγt	BV 421	Q31-378	BD Biosciences	Cat # 562894
TNF	APC	MP6-XT22	Thermo Fischer Scientific	Cat # 17-7321-82
FoxP3	PE	FJK-16s	Thermo Fischer Scientific	Cat # 12-5773-82
hTNF	PE	REA656	Miltenyi Biotec	Cat # 130-118-974

Data were acquired with FACSCanto or LSRII (BD Biosciences) and analysis was performed with FlowJo software (TreeStar).

### ELISA

In order to detect proteins in colonic explant supernatant, enzyme-linked immunosorbent assay (ELISA) for human TNF (Cat #: 88-7346-22), mouse TNF (Cat #: 88-7324-22) and mouse IL-22 (Cat#: 88-7422-22) was performed. All ELISA assays (Ready-Set-Go!) were purchased from ThermoFischer Scientific and performed according to manufacturer´s protocol. Detection of human IL-22BP was done using anti-hIL-22BP (RnDSystems: MAB1087) as capture antibody, biotinylated anti-hIL-22BP (RnDSystems: BAF1087) as detection antibody and rhIL-22BP (RnDSystems: 1087-BP) as a standard. Shortly, Nunc MaxiSorb flat bottom 96-well plates (Nunc) were coated with capture antibody diluted in coating buffer overnight. After washing and blocking with 1x assay diluent, colonic explant samples were diluted 1:2 in assay diluent and applied in duplicates. Bound protein was then detected by a protein-specific biotinylated detection antibody and avidin-horseradish peroxidase and color was developed with TMB substrate solution. Color reaction was stopped with stop solution and plates were scanned with a VMax® Kinetic Microplate reader (Molecular Devices) at respective wavelength (450 nm).

### Multiplex Immunoassay

To assess multiple protein biomarkers simultaneously in colonic explant supernatants, ProcartaPlex Mouse Cytokines and Chemokines (26 plex) (Thermo Fischer Scientific; Cat #: EPX260-26088-901) was performed according to manufacturer’s instructions (Table 3). The plate was read with Luminex Instrument (Luminex) and data were analyzed with ProcartaPlex Analyst Software 1.0 (Thermo Fischer Scientific).

**Table 3:** Antigens detected by ProcartaPlex Mouse Cytokine and Chemokine Panel.

Cytokines	Chemokines
IL-12	MCP-1 (CCL2)
IL-23	RANTES (CCL5)
IL-27	MCP-3 (CCL7)
GM-CSF	GROα (CXCL1)
IFNγ	IP-10 (CXCL10)
IL-1β	Eotaxin
IL-10	MIP-1α
IL-13	MIP-1β
IL-17A	MIP-2
IL-18	
IL-22	
IL-4	
IL-2	
IL-5	
IL-6	
IL-9	
TNF	

### RNA preparation of colonic samples and cells

For RNA isolation, colon pieces were frozen in liquid N_2_ and homogenized with mortar and pestle prior to resuspension in 1 mL of Trizol (Sigma-Aldrich: Cat #: 93289). RNA from cells was isolated by direct addition of Trizol reagent to the pelleted cells. Further, RNA was isolated by phenol-chloroform extraction. Shortly, 200 µL of chloroform was added, samples were shaken thoroughly and centrifuged (13000 rpm for 15 min at 4 °C) and subsequently RNA from the upper aqueous phase was precipitated with 700 µL isopropanol and 200 µg of glycogen to enhance precipitation. After pelleting, RNA was washed with 75 % ethanol and air-dried at 56 °C in a thermoshaker. Depending on the size, the pellet was reconstituted in 10-50 µL of DEPC-treated water (Life Technologies) and dissolved at 56 °C for 10 min. RNA concentration was determined with a NanoDrop photospectrometer (Thermo Scientific).

### Reverse transcription and cDNA synthesis

For reverse transcription, 1 µg of RNA was treated with DNase I (Thermo Fischer Scientific; Cat #: EN0525) according to the manufacturer´s instructions. To transcribe RNA to cDNA, random hexamer primers (TIB Molbiol) and RevertAid H Minus reverse transcriptase (Thermo Fischer Scientific; Cat #: EP0451) were used according to the manufacturer´s protocol. The generated single-stranded cDNA was directly used for quantitative Real-time PCR.

### Quantitative Real-time PCR

cDNA transcript expression of indicated genes (Table 4) was analyzed with Maxima SYBR Green/ROX qPCR master mix (Thermo Fischer Scientific; Cat #: K0222) according to the manufacturer´s protocol by using a StepOne Plus Real Time PCR system (Applied Biosystems) or Mx3000P qPCR system (Stratagene). Reactions were performed in 96-well PCR plates with low profile (Biozym Scientific) and sealed with real-time PCR adhesive seals (Bio-Rad Laboratories). Amplification conditions are listed in Table 5. The relative expression level of each gene was determined by comparing expression to β-actin as a housekeeping gene using the ΔΔCT method.

**Table 4:** Primers for qRT-PCR.

Gene	Primer pair (5‘ > 3‘)
β-actin	CTCCTGAGCGCAAGTACTCTGTG TAAAACGCAGCTCAGTAACAGTCC
IL-22	CATGCAGGA GGTGGTACCTT CAGACGCAAGCATTTCTCAG
IL-22BP	AAGCATTGCCTTCTAGGTCTCC TCAGAGATACACGAGCTGGTT
RegIIIβ	ACTCCCTGAAGAATATACCCTCC CGCTATTGAGCACAGATACGAG
RegIIIγ	ACTTCACCTTGCACCTGAGAA ATGCTTCCCCGTATAACCATCA
Smoothened	GTGCTGTCTACATGCCCAAGT GCAACGCAGAAAGTCAGGC
Axin2	AACCTATGCCCGTTTCCTCTA GAGTGTAAAGACTTGGTCCACC
Ascl2	AAGCACACCTTGACTGGTACG AAGTGGACGTTTGCACCTTCA
Lgr5	CCTACTCGAAGACTTACCCAGT GCATTGGGGTGAATGATAGCA
CD44	CCCAGTGACCCCTGCTAAAA CGCACTTGAGTGTCCAGCTA
Survivin	GAGGCTGGCTTCATCCACTG CTTTTTGCTTGTTGTTGGTCTCC
Human β-actin	AGAGCTACGAGCTGCCTGAC AGCACTGTGTTGGCGTACAG
Human IL-22BP	TGTTGGGGTACTCAAGAACTCT CCCTCCCGTAATAAGGTTCCTG

**Table 5:** Conditions for qRT-PCR.

Step	Temperature	Time
Initial activation step	95 °C	10 min
50 cycles	95 °C	15 sec
	60 °C	30 sec
	72 °C	30 sec
	95 °C	1 min (with gradient of the temperature - 1,6 C/s)
	55 °C	30 sec (with gradient of the temperature - 0,05 C/s
	95 °C	30 sec

### Statistical analysis

Statistics were calculated in Graph Pad Prism (GraphPad Software). Statistical significance was determined using Student´s t-test for unpaired data and statistical significance was defined as follows: *p < 0.05, **p < 0.01, ***p < 0.001. Statistically evaluated values are expressed as mean with error bars representing standard deviation from mean (SEM). The Pearson correlation was used for correlative analyses. The significance level was set to *p* ≤ 0.05.

## Supplementary information

Supplementary information

Supplementary Table
