# Supplementary material for: TNF hampers intestinal tissue repair in colitis by restricting IL-22 bioavailability
Source: Mucosal Immunol. 2022 Apr 5;15(4):698–716. doi: 10.1038/s41385-022-00506-x (PMC9259490; doi:10.1038/s41385-022-00506-x)
Supplement: Supplementary file 1 — Supplementary information [file 41385_2022_506_MOESM1_ESM.pdf]

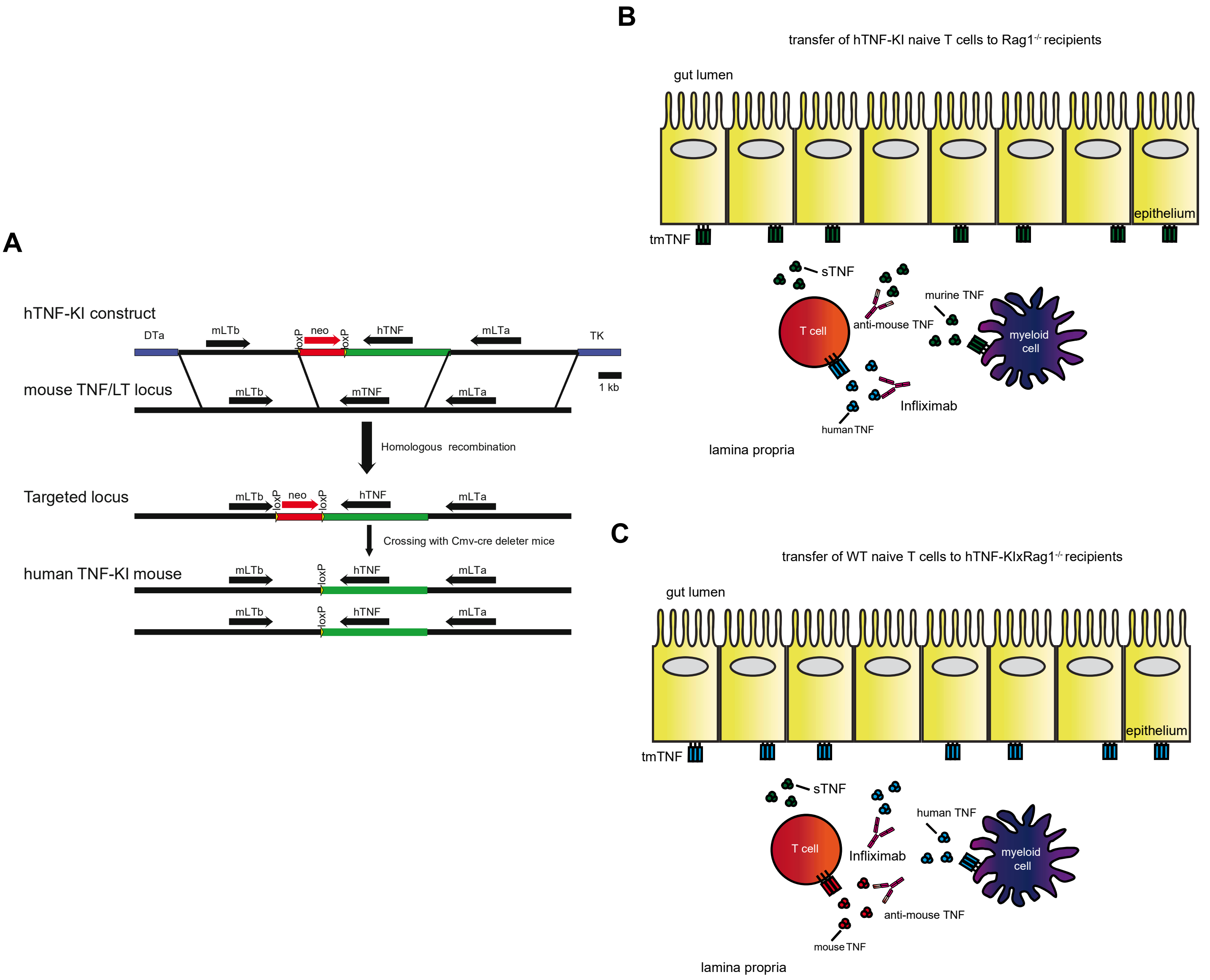


**Figure S1. Inhibition of disease by human TNF blockade in humanized TNF model of colitis.** (**A**) Scheme of human TNF knock-in (hTNF-KI) mice. (**B**) Scheme of T cell-derived TNF inhibition in humanized TNF colitis model. Rag1$^{-/-}$ were reconstituted with naive T cells from hTNF-KI mice. Mice were treated once they lost > 5 % of their initial weight twice per week for two or three weeks with various anti-TNF agents or respective controls (10 mg/kg; i.p.). Utilisation of infliximab allows blocking only T cell-derived TNF, administration of anti-mTNF blocks TNF produced by non-T cells. (**C**) Scheme of non-T cell-derived TNF inhibition in humanized TNF colitis model. hTNF-KIxRag1$^{-/-}$ were reconstituted with naive T cells from WT mice. Mice were treated once they lost > 5 % of their initial weight twice per week for two or three weeks with various anti-TNF agents or respective controls (10 mg/kg; i.p.). Utilisation of infliximab allows blocking only non-T cell-derived TNF, administration of anti-mTNF blocks TNF produced by T cells.

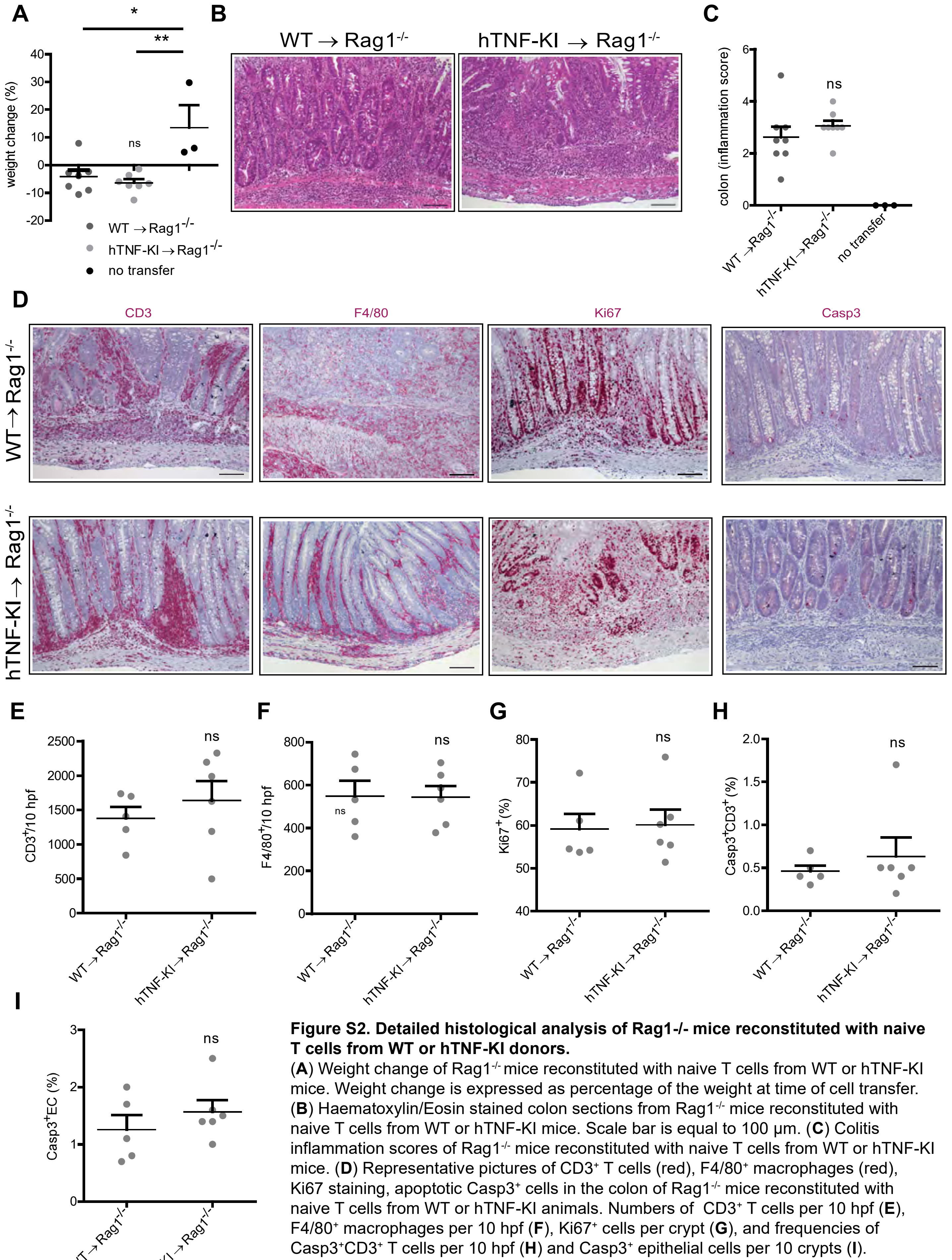


**Figure S2. Detailed histological analysis of Rag1-/- mice reconstituted with naive T cells from WT or hTNF-KI donors.**

(**A**) Weight change of Rag1$^{-/-}$ mice reconstituted with naive T cells from WT or hTNF-KI mice. Weight change is expressed as percentage of the weight at time of cell transfer. (**B**) Haematoxylin/Eosin stained colon sections from Rag1$^{-/-}$ mice reconstituted with naive T cells from WT or hTNF-KI mice. Scale bar is equal to 100 μm. (**C**) Colitis inflammation scores of Rag1$^{-/-}$ mice reconstituted with naive T cells from WT or hTNF-KI mice. (**D**) Representative pictures of CD3$^{+}$ T cells (red), F4/80$^{+}$ macrophages (red), Ki67 staining, apoptotic Casp3$^{+}$ cells in the colon of Rag1$^{-/-}$ mice reconstituted with naive T cells from WT or hTNF-KI animals. Numbers of CD3$^{+}$ T cells per 10 hpf (**E**), F4/80$^{+}$ macrophages per 10 hpf (**F**), Ki67$^{+}$ cells per crypt (**G**), and frequencies of Casp3$^{+}$CD3$^{+}$ T cells per 10 hpf (**H**) and Casp3$^{+}$ epithelial cells per 10 crypts (**I**).
All data are representative of two or more independent experiments.
Data represent mean values + SEM. *, p<0.05, **, p<0.01, ***, p<0.001, as calculated by Student's t-test; ns, not significant. Hpf, high power field.

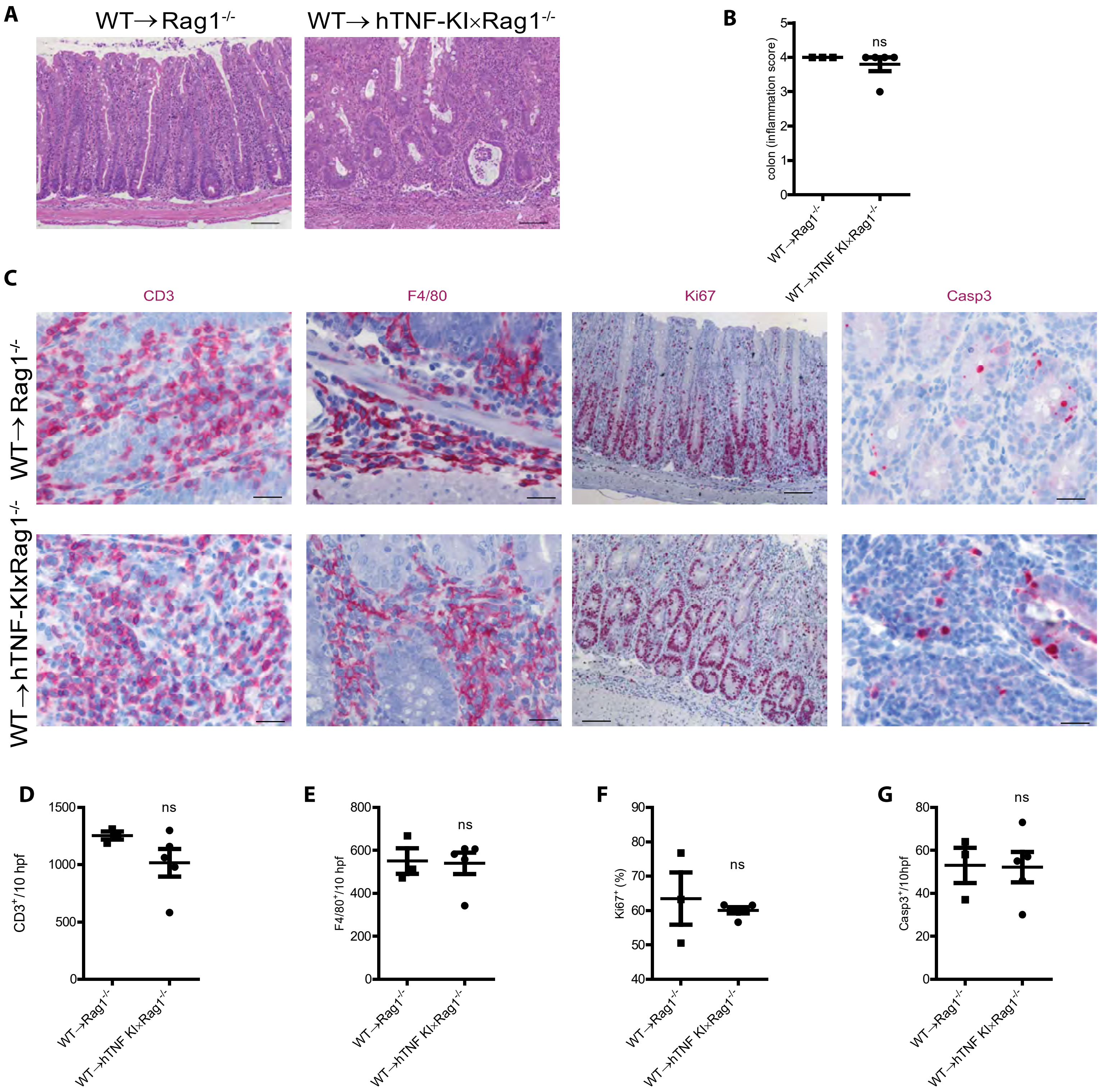


**Figure S3. Detailed histological analysis of Rag1$^{-/-}$ and hTNF-KIxRag1$^{-/-}$ mice reconstituted with naive T cells from WT donors.**

(**A**) Haematoxylin/Eosin stained colon sections from Rag1$^{-/-}$ and hTNF-KIxRag1$^{-/-}$ mice reconstituted with naive T cells from WT mice. Scale bar is equal to 100 µm. (**B**) Colitis inflammation scores of Rag1$^{-/-}$ and hTNF-KIxRag1$^{-/-}$ mice reconstituted with naive T cells from WT mice. (**C**) Representative pictures of CD3$^{+}$ T cells (red), F4/80$^{+}$ macrophages (red), Ki67 staining, apoptotic Casp3$^{+}$ cells in the colon of Rag1$^{-/-}$ and hTNF-KIxRag1$^{-/-}$ mice reconstituted with naive T cells from WT animals. Numbers of CD3$^{+}$ T cells per 10 hpf (**D**), F4/80$^{+}$ macrophages per 10 hpf (**E**), Ki67$^{+}$ cells per crypt (**F**) and numbers of Casp3$^{+}$ cells per 10 hpf (**G**). All data are representative of two independent experiments. Data represent mean values + SEM. *, $p<0.05$, **, $p<0.01$, ***, $p<0.001$, as calculated by Student's t-test; ns, not significant. Hpf, high power field.

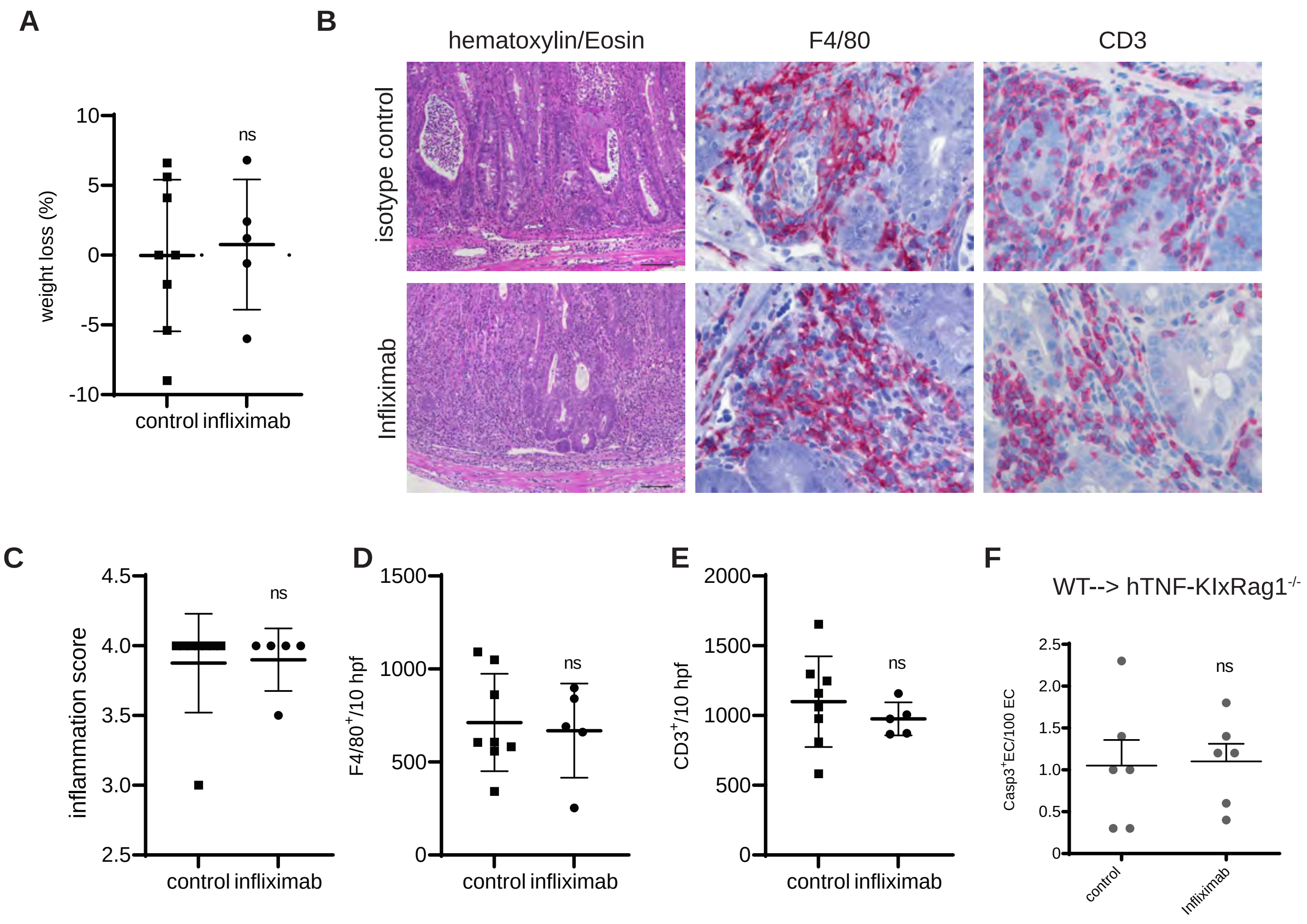


**Figure S4. Dispensable role of non-T cell-derived TNF in established humanized TNF colitis model.** Naive WT T cells were transferred to hTNFK-KI×Rag1$^{-/-}$ recipients, anti-TNF (infliximab; 10 mg/kg; i.p. twice per week) was administered once mice have lost 5% of their initial weight. **(A)** Weight changes 3 weeks after treatment of colitic mice with either Fc control or infliximab (both 10mg/kg; i.p, twice per week). **(B)** Representative images of Hematoxylin/Eosin (left panel), F4/80$^{+}$ macrophages (middle panel) and CD3$^{+}$ T cells (red) (right panel) stained tissue sections of the colon in mice treated with either Fc control or infliximab for 3 weeks. Inflammation score **(C),** F4/80$^{+}$ macrophages per 10 hpf (**D**) CD3$^{+}$ T cells per 10 hpf (**E**) of the colon in mice treated with either Fc control or infliximab for 3 weeks.
(**F**) Casp3$^{+}$ EC cells per 100 EC of the colon in mice treated with either Fc control or infliximab for 2 weeks. Data are representative of two ndependent experiments. Data represent mean values ± SEM.

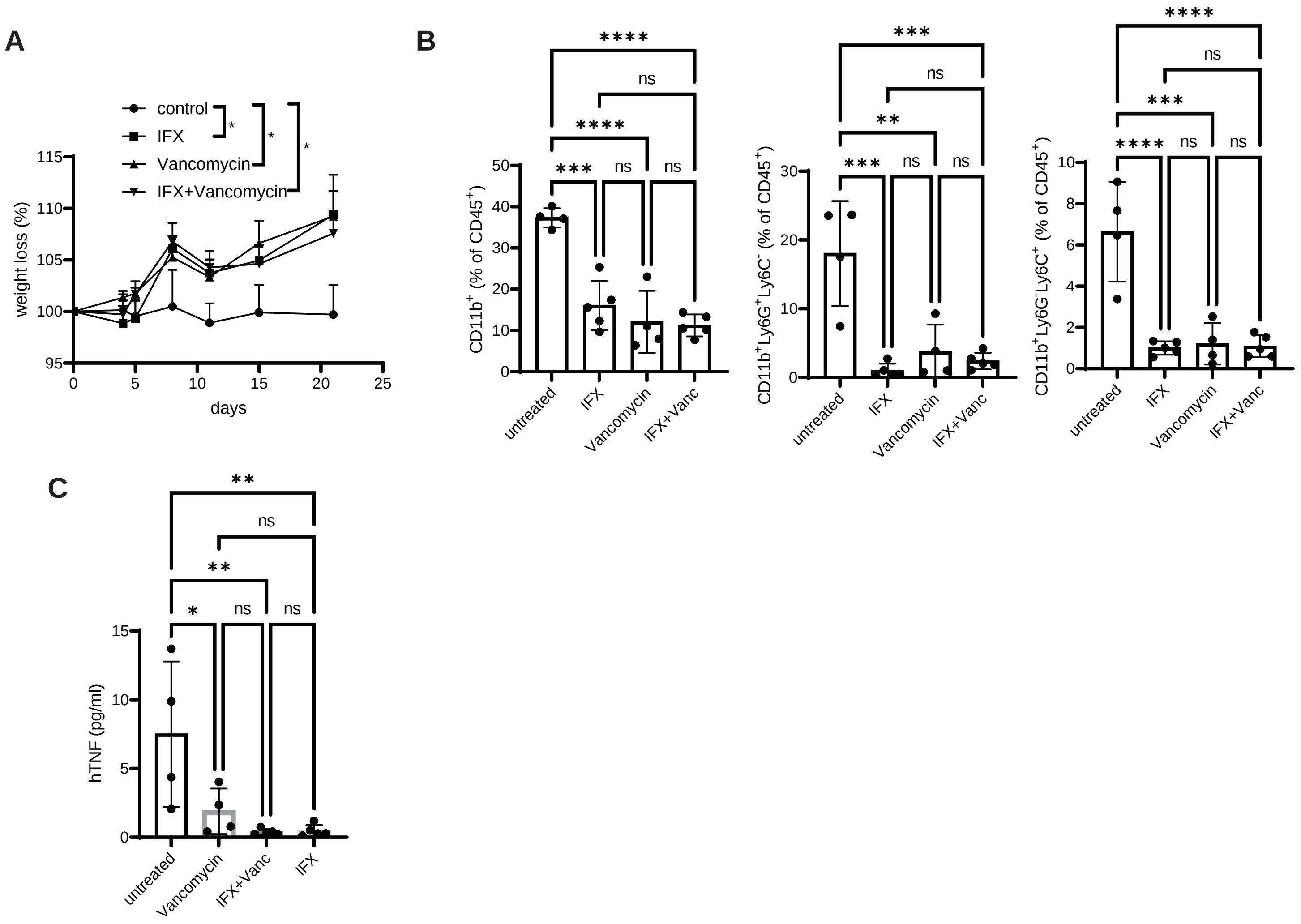


**Figure S5. Effect of Vancomycin treatment on anti-TNF therapy during colitis.**
Naive hTNF KI T cells were transferred to Rag1$^{-/-}$ recipients, anti-TNF (infliximab; 10 mg/kg; i.p. twice per week) or Fc control (10 mg/kg; i.p. twice per week) was administered once mice have lost 5% of their initial weight. Simultaneously, mouse microbiota was modified by provision of Vancomycin (0,25 mg/ml) in drinking water. **(A)** Weight loss of mice treated with infliximab or Fc control. (**B**) Frequencies of CD11b$^+$ cells, inflammatory monocytes (CD45$^+$CD11b$^+$Ly6G$^-$Ly6C$^+$) and  granulocytes (CD45$^+$CD11b$^+$Ly6G$^+$Ly6C$^-$) in colons 3 weeks after therapy. (**C**) Levels of hTNF in ex vivo colonic explants from mice treated with for 3 weeks. Data are representative of two independent experiments.
Data represent mean values ± SEM.

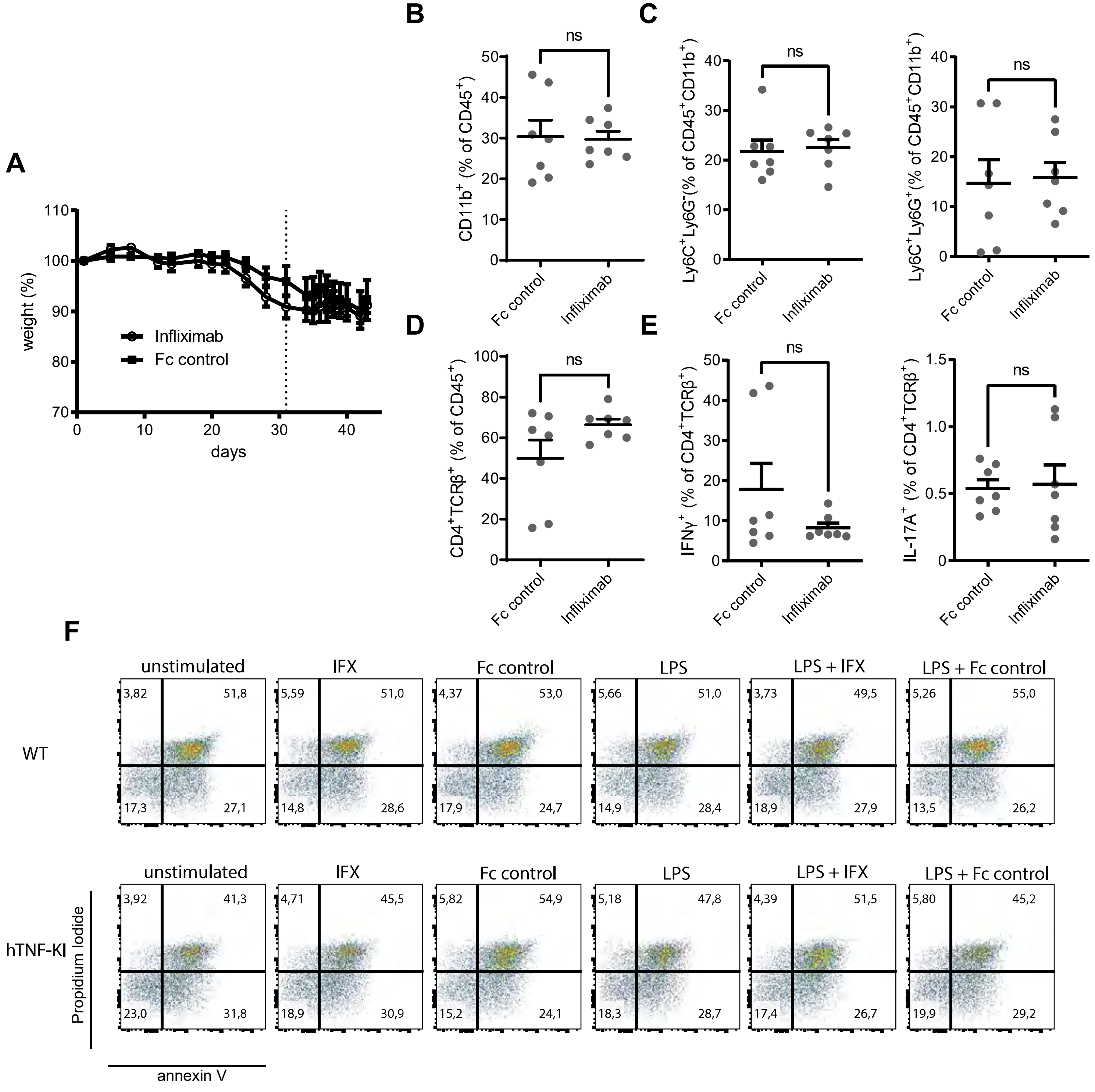


**Figure S6. TNF independent effects of infliximab treatment during colitis.** Naive WT T cells were transferred to Rag1$^{-/-}$ recipients, anti-TNF (infliximab; 10 mg/kg; i.p. twice per week) or Fc control (10 mg/kg; i.p. twice per week) was administered once mice have lost 5% of their initial weight. **(A)** Weight loss of mice treated with infliximab or Fc control, dashed line indicates start of the treatment. Frequencies of CD11b+ cells (B), inflammatory monocytes ($CD45^+CD11b^+Ly6G^-Ly6C^+$) and granulocytes ($CD45^+CD11b^+Ly6G^+Ly6C^+$) (**C**) in the colon 2 weeks after therapy. Frequencies of T cells (**D**), Th1 ($CD45^+CD4^+TCR\beta^+IFN\gamma^+$) and Th17 ($CD45^+CD4^+TCR\beta^+IL\text{-}17A^+$) (**E**) in the colon 2 weeks after therapy. (**F**) Effect of infliximab on the survival of splenocytes in vitro upon LPS treatment. Data are representative of two independent experiments. Data represent mean values ± SEM.

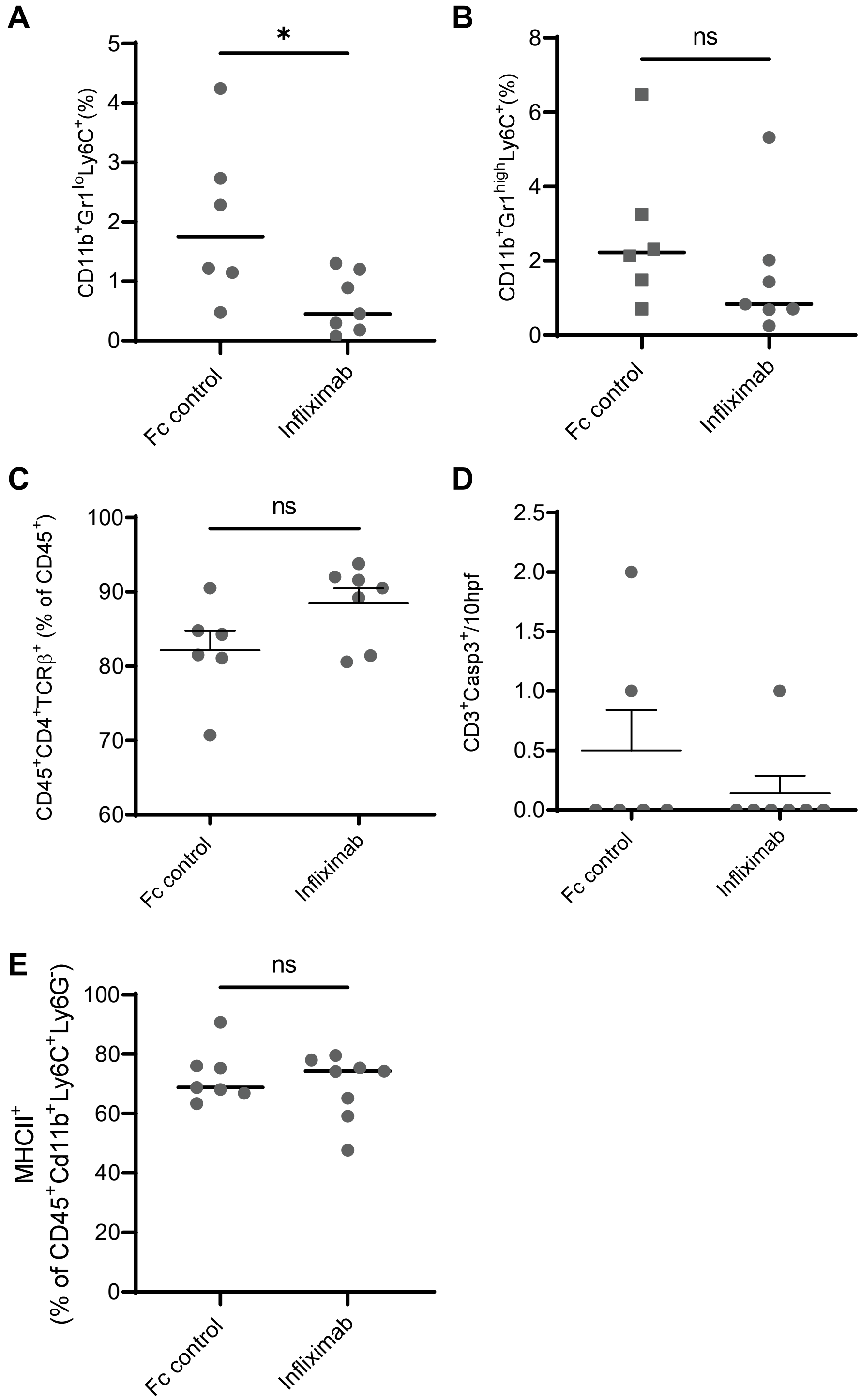


**Figure S7. T cell-derived TNF is pathogenic during established colitis.**

Naive hTNF-KI T cells were transferred to Rag1$^{-/-}$ recipients, anti-TNF (infliximab; 10 mg/kg; i.p. twice per week) was administered once mice lost 5% of their initial weight.

(**A**, **B**) Frequencies of granulocytes ($CD45^+CD11b^+Gr1^{high}Ly6C^+$) and inflammatory monocytes ($CD45^+CD11b^+Gr1^{low}Ly6C^+$) in the mesenteric lymph node 2 weeks after anti-TNF therapy.

(**C**) Frequencies of T cells ($CD45^+CD4^+TCR\beta^+$) in the colon 2 weeks after anti-TNF therapy.

(**D**) Numbers of $CD3^+Casp3^+$ T cells per 10 hpf in the colon 2 weeks after anti-TNF therapy.

(**E**) Frequencies of MHCII-expressing monocytes ($CD45^+CD11b^+Ly6G^-Ly6C^+MHCII^+$) in colons 2 weeks after anti-TNF therapy.

All data are representative of two independent experiments.

Data represent mean values ± SEM. *, $p<0.05$, **, $p<0.01$, ***, $p<0.001$, as calculated by Student's t-test; ns, not significant.

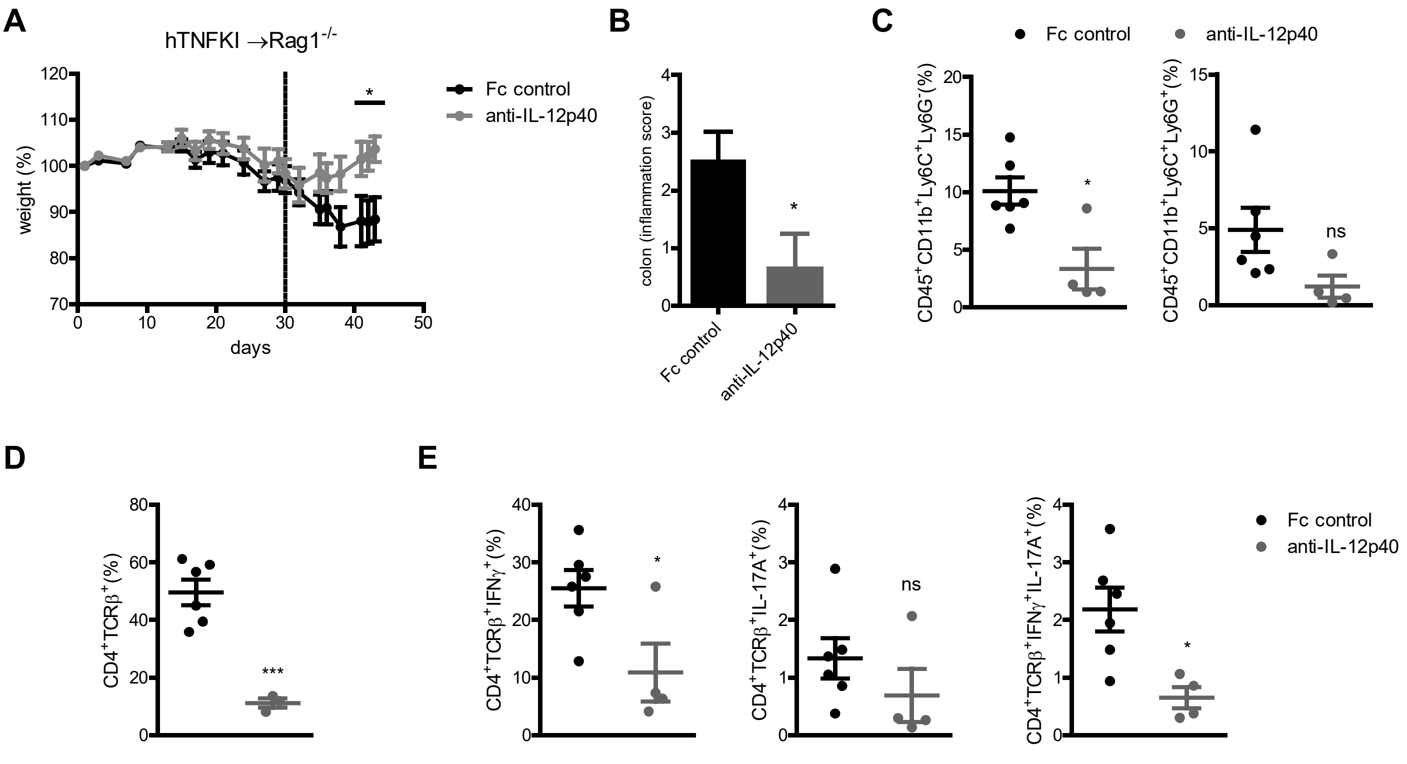


**Figure S8. Characterization of colitis induced by transfer of hTNF-KI naive T cells into Rag1$^{-/-}$ recipients.** Weight (**A**) and inflammation score (**B**) of Rag1$^{-/-}$ animals injected with CD4$^+$CD25$^-$CD45RB$^{high}$ cells from hTNF-KI mice and treated either with anti-IL-12p40 (clone C17.8; 10 mg/kg, i.p.; twice per week) or respective isotype control. (**C**) Frequencies of granulocytes (CD45$^+$CD11b$^+$Ly6G$^+$Ly6C$^+$) and inflammatory monocytes (CD45+CD11b+Ly6G-Ly6C+) in the colon after 2 weeks of anti-IL-12p40 therapy. (D) Frequencies of CD4 T cells (CD45$^+$CD4$^+$TCRβ$^+$) after 2 weeks of anti-IL-12p40 therapy. (**E**) Frequencies of Th1 (CD45$^+$CD4$^+$TCRβ$^+$IFNγ$^+$), Th17 (CD45$^+$CD4$^+$TCRβ$^+$IL-17A$^+$) and Th1/17 (CD45$^+$CD4$^+$TCRβ$^+$IL-17A$^+$IFNγ$^+$) cells after 2 weeks of anti-IL-12p40 therapy. All data are representative of two independent experiments. Data represent mean values ± SEM. *, $p<0.05$, **, $p<0.01$, ***, $p<0.001$, as calculated by Student's t-test; ns, not significant.

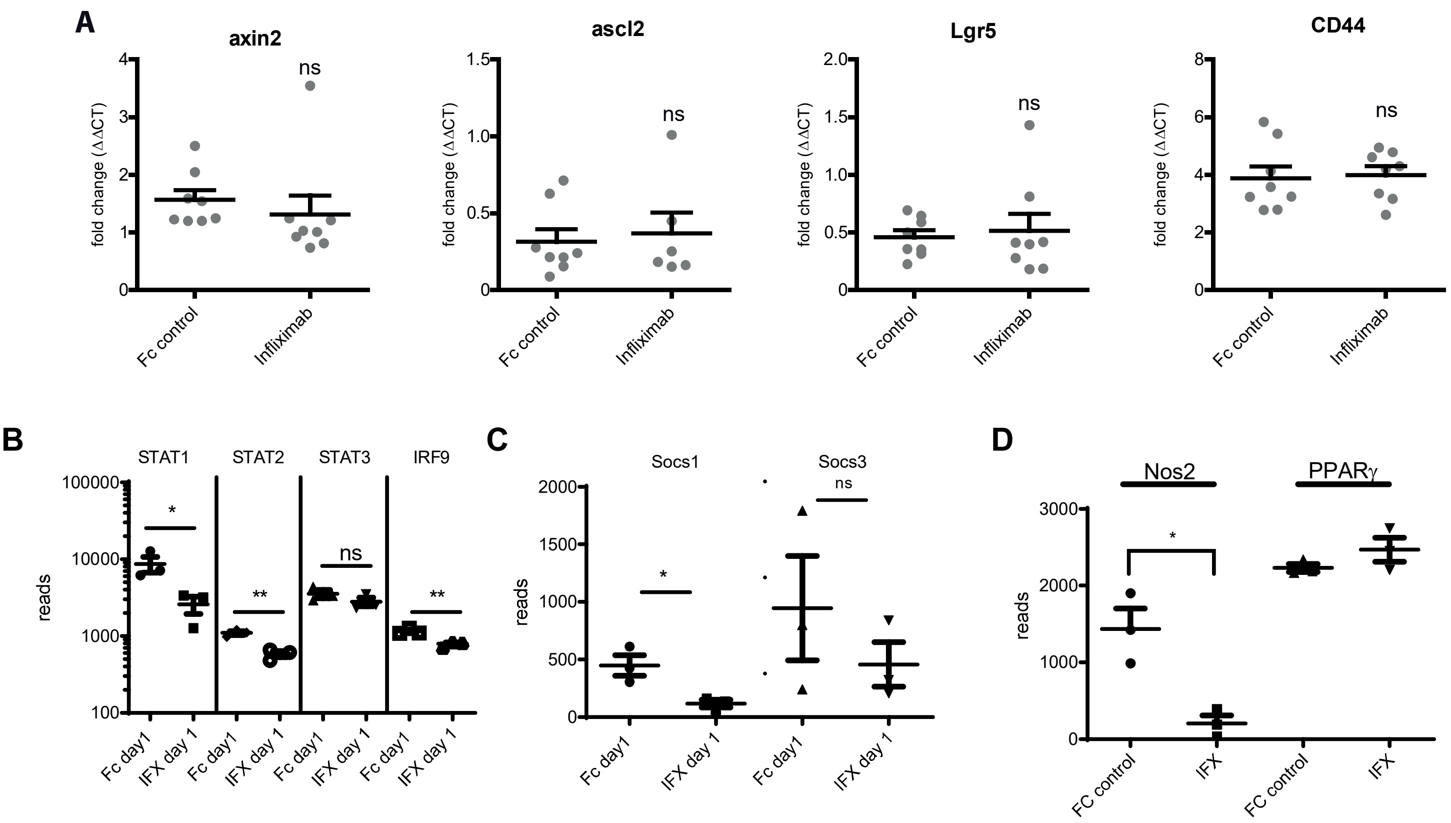


**Figure S9. Analysis of colonic epithelial cells during anti-TNF therapy during colitis.** (**A**) Expression of WNT dependent genes (ascl2, Lgr5, CD44, axin2) in the colon after 2 weeks of anti-TNF therapy. (**C**, **D**, **E**). Expression of selected genes in in colonic EC 24 hours after infliximab treatment, when compared to Fc-control treated group.
Student's T-test, * - p<0,05, ** - p<0,005,*** - p<0,005, **** - p<0,0001, n.s. – not significant

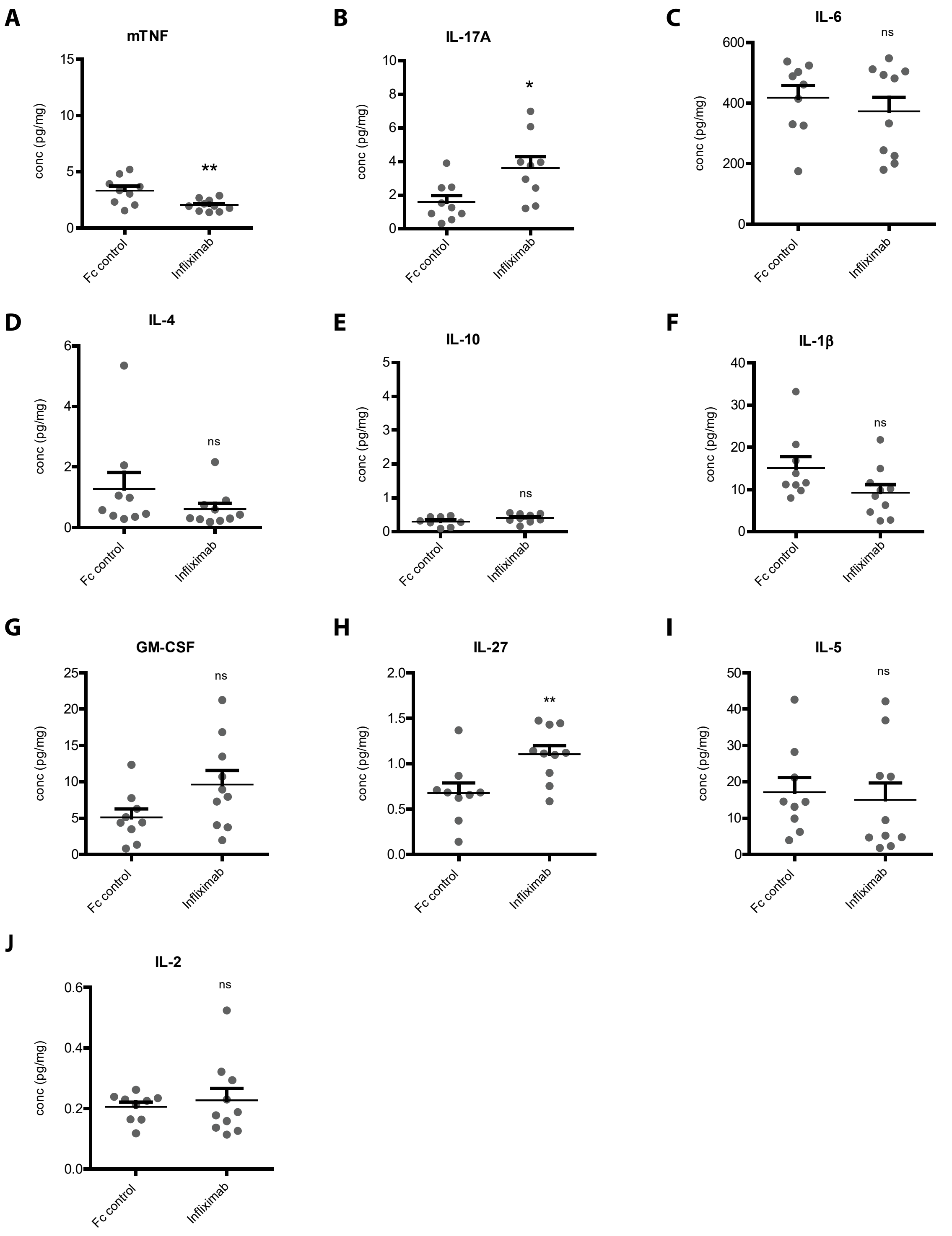


**Figure S10. Cytokine expression in the colon upon T-TNF blockade during established colitis.** Expression of murine TNF (**A**), IL-17A (**B**), IL-6 (**C**), IL-4(**D**), IL-10 (**E**), IL-1β (**F**), GM-CSF (**G**), IL-27 (**H**), IL-5 (**I**) and IL-2 (**J**) in colonic explant supernatants (24 hrs) of Rag1$^{-/-}$ mice reconstituted with naive T cells from hTNF-KI mice and treated with either infliximab or Fc control for 2 weeks (10mg/kg; i.p. twice per week) was measured with Multiplex Immunoassay and concentrations were calculated per mg of colonic tissue (pg/mg). All data are representative of two or more independent experiments with n≥3.
Data represent mean values + SEM. *, p<0.05, **, p<0.01, ***, p<0.001, as calculated by Student's t-test; ns, not significant.

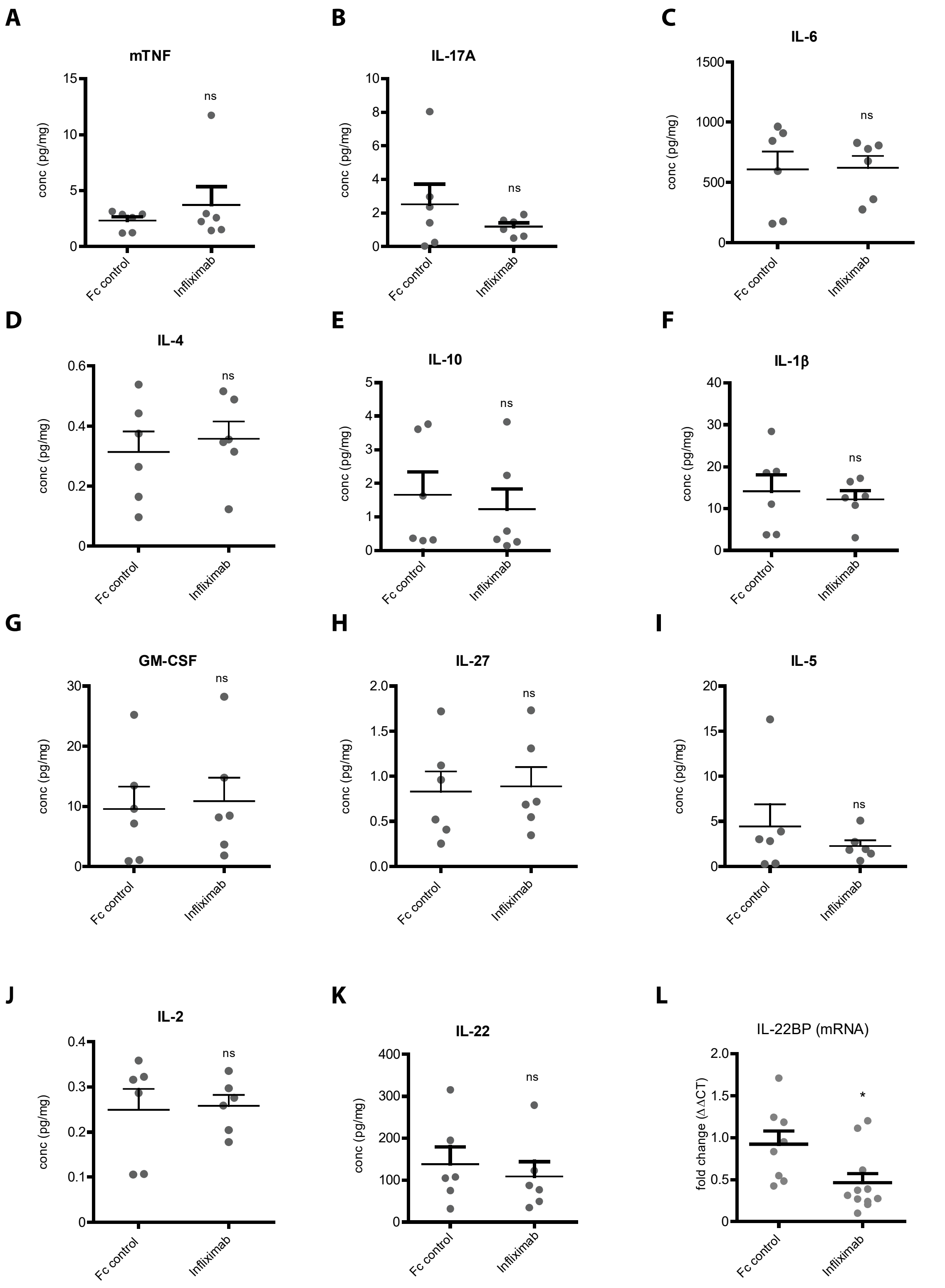


**Figure S11. Cytokine expression in the colon upon "non-T"-TNF blockade during established colitis.** Expression of murine TNF (**A**), IL-17A (**B**), IL-6 (**C**), IL-4 (**D**), IL-10 (**E**), IL-1β (**F**), GM-CSF (**G**), IL-27 (**H**), IL-5 (**I**), IL-2 (**J**) and IL-22 (**K**) in colonic explant supernatants (24 hrs) of hTNF-KIxRag1$^{-/-}$ mice reconstituted with naive WT T cells and treated with either infliximab or Fc control for 2 weeks (10mg/kg; i.p. twice per week) was measured with Multiplex Immunoassay and concentrations were calculated per mg of colonic tissue (pg/mg). (**L**) IL-22BP mRNA levels in the colons of mice treated with Fc control or infliximab for 2 weeks. Data from two experiments were pooled.
All data are representative of two or more independent experiments with n≥3.
Data represent mean values + SEM. *, $p<0.05$, **, $p<0.01$, ***, $p<0.001$, as calculated by Student's t-test; ns, not significant.

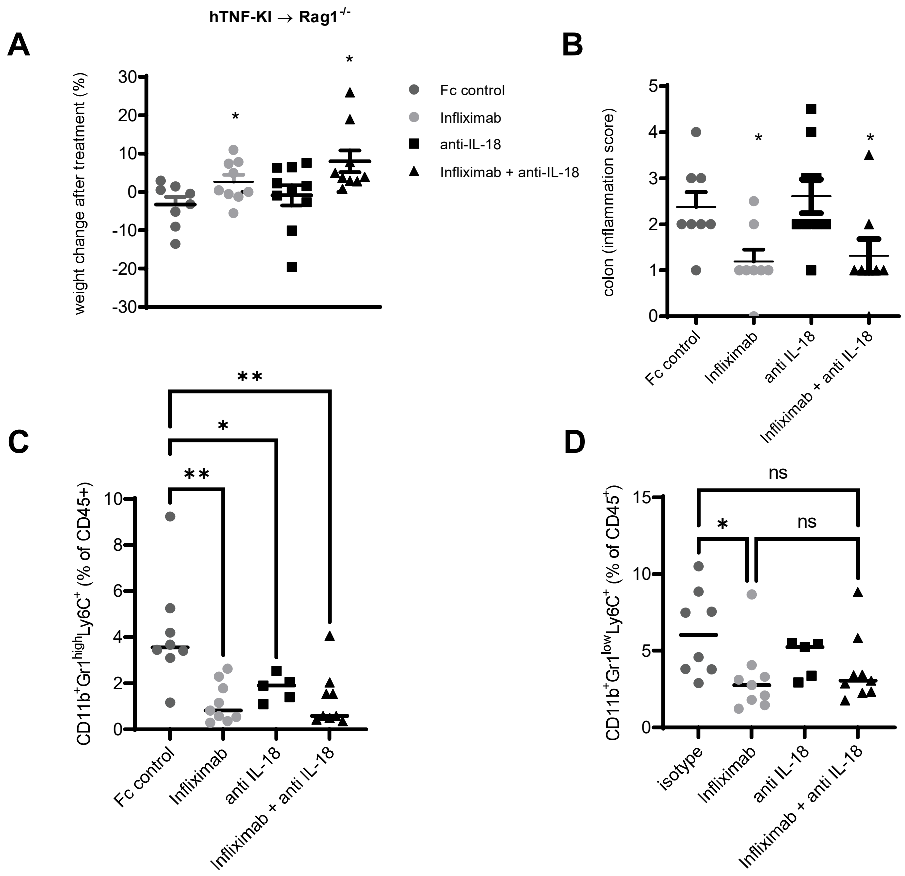


**Figure S12. Role of IL-18 in anti-TNF induced recovery from colitis in humanized TNF colitis model.**

Naive hTNF KI T cells were transferred to Rag1-/- recipients, anti-TNF (infliximab; 10 mg/kg; i.p. twice per week); IL-18 (10mg/kg; i.p. twice per week), Fc control (10 mg/kg; i.p. twice per week) or infliximab/anti-IL-18 (both 10mg/kg; i.p. twice per week) was administered once mice have lost 5% of their initial weight. **(A)** Weight changes 3 weeks after treatment of colitic mice. **(B)** inflammation score of the colon in mice treated for 3 weeks.Frequencies of granulocytes ($CD45^+CD11b^+Gr1^{high}Ly6C^+$) (**C**) and inflammatory monocytes ($CD45^+CD11b^+Gr1^{low}Ly6C^{+)}$ (**D**) in the colon 3 weeks after therapy. Data are representative of two independent experiments.

Data represent mean values ± SEM.

-

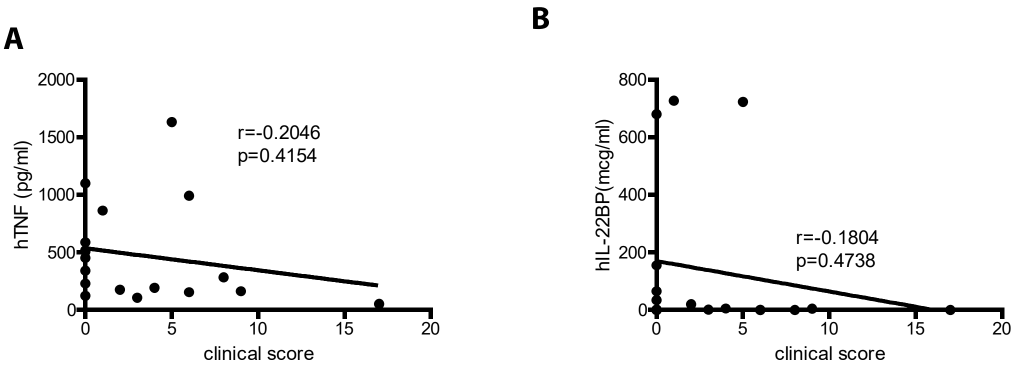


**Figure S13. Correlation of serum cytokine levels and clinical score in IBD patients.**
(**A**) Correlation between hTNF levels and clinical score in IBD patients.
(**B**) Correlation between hIL-22BP levels and clinical score in IBD patients.
The Pearson correlation was used for correlative analyses. The significance level was set to $p < 0.05$.

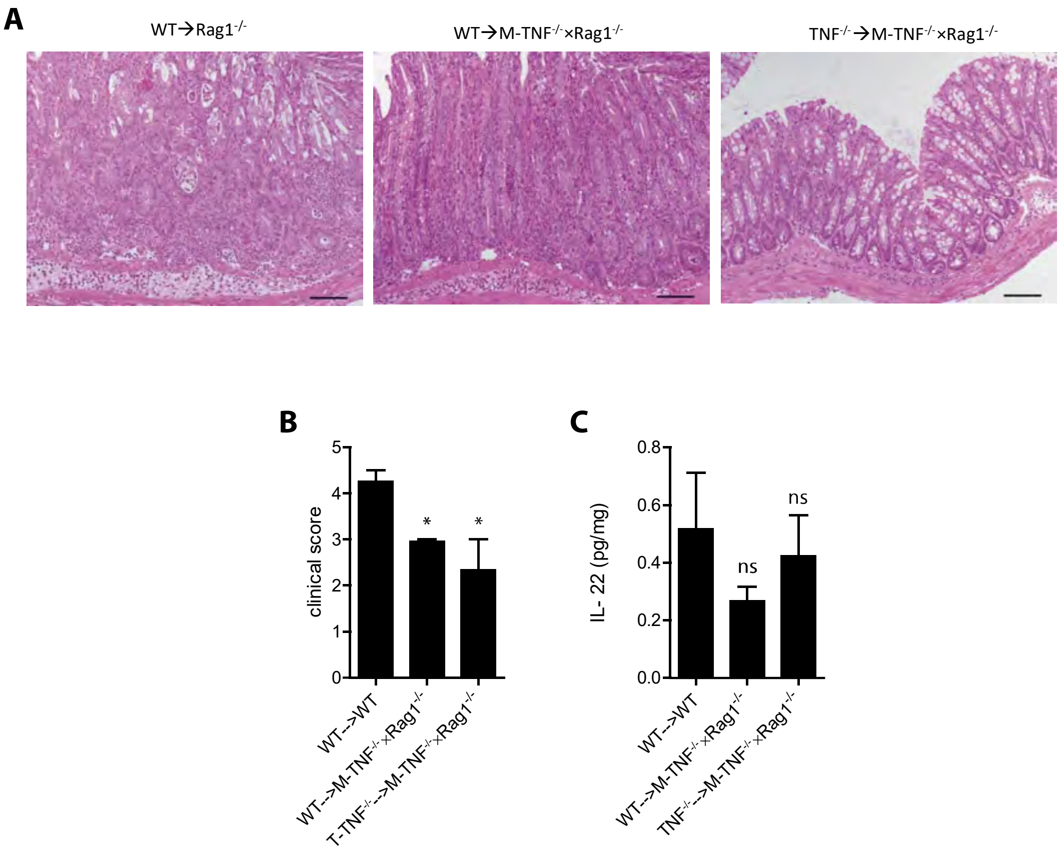


**Figure S14. Contribution of individual cellular sources of TNF to the epithelial cell proliferation and IL-22BP expression in the colon. A**. Representative pictures of colonic tissue sections of Rag1$^{-/-}$, M-TNF$^{-/-}$×Rag1$^{-/-}$ reconstituted with naive T cells from WT donors and M-TNF$^{-/-}$×Rag1$^{-/-}$ mice reconstituted with naive T cells from TNF$^{-/-}$ donors stained with Hematoxylin/Eosin. **B**. Colonic inflammation score of Rag1$^{-/-}$, M-TNF$^{-/-}$×Rag1$^{-/-}$ reconstituted with naive T cells from WT donors and M-TNF$^{-/-}$×Rag1$^{-/-}$ mice reconstituted with naive T cells from TNF$^{-/-}$ donors. **C.** IL-22 levels in colonic explants of Rag1$^{-/-}$, M-TNF$^{-/-}$×Rag1$^{-/-}$ reconstituted with naive T cells from WT donors and M-TNF$^{-/-}$×Rag1$^{-/-}$ mice reconstituted with naive T cells from TNF$^{-/-}$ donors.
